# Correction: Effects of a low-carbohydrate diet in adults with type 1 diabetes management: A single arm non-randomised clinical trial

**DOI:** 10.1371/journal.pone.0329632

**Published:** 2025-08-01

**Authors:** Jessica L. Turton, Grant D. Brinkworth, Helen M. Parker, David Lim, Kevin Lee, Amy Rush, Rebecca Johnson, Kieron B. Rooney

The word “management” should be removed in the article title. The correct title is: Effects of a low-carbohydrate diet in adults with type 1 diabetes: A single arm non-randomised clinical trial. The correct citation is: Turton JL, Brinkworth GD, Parker HM, Lim D, Lee K, Rush A, et al. (2023) Effects of a low-carbohydrate diet in adults with type 1 diabetes: A single arm non-randomised clinical trial. PLoS ONE 18(7): e0288440. https://doi.org/10.1371/journal.pone.0288440
