# Supplementary material for: Effects of a low-carbohydrate diet in adults with type 1 diabetes management: A single arm non-randomised clinical trial
Source: PLoS One. 2023 Jul 11;18(7):e0288440. doi: 10.1371/journal.pone.0288440 (PMC10335683; doi:10.1371/journal.pone.0288440)
Supplement: S1 File — (PDF) [file pone.0288440.s001.pdf]

Study Title: Effects of a Low-Carbohydrate Diet in Adults with Type 1 Diabetes: An Interventional Study

Short title: A Novel Dietary Approach for Adults with Type 1 Diabetes

STATEMENT OF COMPLIANCE FOR NON DRUG OR DEVICE CLINICAL TRIALS

This document is a protocol for a clinical research study. The study will be conducted in compliance with all stipulations of this protocol, the conditions of ethics committee approval, the NHMRC National Statement on Ethical Conduct in Human Research (as updated) and the Handbook for Good Clinical Research Practice (GCP). The Therapeutic Goods Act has adopted ICH Guideline for Good Clinical Practice.

Contents

1. GENERAL INFORMATION 3
2. SYNOPSIS 4
3. RATIONALE / BACKGROUND 5
4. AIMS / OBJECTIVES / HYPOTHESES 8
5. PARTICIPATING SITES 9
6. RESEARCH PLAN / STUDY DESIGN 9
7. ETHICAL CONSIDERATIONS 16
8. SAFETY CONSIDERATIONS 17
9. OUTCOMES 18
10. DATA MANAGEMENT 29
11. TIMELINES / MILESTONES 31
12. FINANCIAL 32
13. PUBLICATION POLICY / DISSEMINATION OF RESULTS 32
14. REFERENCES 33
15. APPENDICES 25

1. GENERAL INFORMATION

1.1 Protocol Title

Effects of a Low-Carbohydrate Diet in Adults with Type 1 Diabetes: An Interventional Study *Protocol* (19/02/2020)

1.2 Study Sponsor

The University of Sydney, Camperdown NSW 2006.

1.3 Name of the Study Funder

This study has been partially funded by The Commonwealth Scientific and Industrial Research Organisation. Ms Turton has also raised money through philanthropic donations and plans to apply for any additional funding that may be required from the University of Sydney under the Postgraduate Research Support Scheme (PRSS) in 2021.

Medtronic Australasia will provide the Continuous Blood Glucose Monitoring sensors and devices for all participants throughout the study.

The Type 1 Diabetes Family Centre have agreed to donate the necessary funds to cover the study diabetes educator's role.

1.4 Investigators Responsible for Conducting the Research

Ms Jessica Turton
A/Prof Kieron Rooney
Prof Grant Brinkworth
Dr Helen Parker
Dr Kevin Lee
Dr David Lim
Mrs Amy Rush
Ms Rebecca Johnson

1.5 Institutions Involved in the Research

The University of Sydney, Camperdown NSW 2006.

1.6 Trial Registration

This clinical trial has been registered with the Australian New Zealand Clinical Trials Registry (ANZCTR) http://www.anzctr.org.au/ (Clinical Trial Number: ACTRN12621000764831).

2. SYNOPSIS

**TITLE**	Effects of a Low-Carbohydrate Diet in Adults with Type 1 Diabetes: An Interventional Study
**OBJECTIVES**	The aim of this research is to assess the efficacy of a low-carbohydrate (LC) diet in adults with type 1 diabetes (T1D). We will specifically set out to determine whether significant differences in T1D management outcomes exist between a LC diet and habitual diets higher in carbohydrate.
**PRIMARY HYPOTHESIS**	A low-carbohydrate (LC) diet will result in improved type 1 diabetes management compared to habitual diets higher in carbohydrates.
**DESIGN**	This is a 28-week single arm within-participant intervention study involving a 4-week control period, a 12-week intervention period and a 12-week follow-up.
**BLINDING/MASKING**	Since our intervention involves the manipulation of dietary carbohydrates and carbohydrate counting is integral for calculating insulin requirements in T1D management, it is not possible or recommended to blind participants in the intervention. The dietitian delivering the intervention will also be completing data collection and analysis. As such, the outcome assessor will also not be blinded to the intervention.
**OUTCOMES**	The primary outcome is haemoglobin A1c (HbA1c) and secondary outcomes include glycaemic variability, frequency of hypoglycaemia, total daily insulin, and quality of life.
**STUDY DURATION**	12 months
**INTERVENTION/S**	This study will investigate a LC diet with an adaptive carbohydrate prescription that starts at 50 g of digestible carbohydrate per day and is then adapted (increased or decreased) within a broader range of 25-75 g/day according to individual blood glucose levels and personal preference. Participants will meet individually with the study dietitian for a total of six fortnightly sessions to receive dietary instruction, strategies and education.
**NUMBER OF PARTICIPANTS**	20
**POPULATION**	Adults (18-70 yrs) with T1D
**SELECTION AND ENROLMENT**	**Inclusion criteria**<br>• Adults aged 18-70 yrs<br>• Residing within Australia<br>• Previously diagnosed T1D (confirmed with specialist letter/correspondence, diabetes duration ≥6 months, using multiple daily injections of insulin and/or insulin pump)<br>• Suboptimal glycaemic control (HbA1c >7.0%)<br>• BMI 18.5-39.9 kg/m^2<br>• Habitual dietary intake contains >150 g/day of total carbohydrates (assessed by study dietitian)<br>• Independent free-living, able to understand study requirements, speak and understand fluent English, and

	both physically and cognitively able to provide their informed consent<br>• Be willing to self-monitor blood glucose levels using both finger-prick and/or continuous/flash blood glucose monitoring devices on a daily basis<br>• Be willing to learn and utilise dietary carbohydrate and protein counting skills<br>• Be willing to titrate insulin dosages according to their dietary carbohydrate (+/- protein) intake, in consultation with a member of their usual care team<br>• Owns and can competently use an iOS or Android mobile device that is able to receive text messages/calls and download free mobile applications from the *App Store* or *Google Play*<br>• Has access to and can competently use a computer, mobile or tablet device with internet connection for uploading data onto REDCap and for Zoom video conferencing<br>• Be willing to visit an Australian Clinical Labs collection centre (https://www.clinicallabs.com.au/location/) to have bloods drawn on four separate occasions throughout the study<br><br>**Exclusion criteria**<br>• HbA1c ≤7.0%<br>• Underweight (BMI <18.5 kg/m^2) or class III obesity (BMI ≥40 kg/m^2)<br>• Habitual use of an automated insulin delivery system to control blood glucose levels<br>• Habitual adherence to a fixed insulin regimen such that they are not confident in titrating insulin dosages according to their dietary intake<br><br>• Previously diagnosed hypo unawareness<br>• Non-English speaking or unable to understand English<br>• Previous medical diagnosis of cognitive impairment and/or mental illness (not including depression and anxiety)<br>• Using medication to manage depression and/or anxiety and medications have been increased within the last 3 months<br>• Current physical impairment or disability that limits individual capacity to communicate with researchers and/or use the mobile application *Easy Diet Diary* (e.g., blind/vision impaired/deaf/speech impaired)<br>• Habitual dietary intake contains ≤150 g/day of total carbohydrates (assessed by study dietitian)<br>• Usual dietary intake strictly excludes animal-based protein (e.g., vegan diet)<br>• Undertaken major change(s) to their insulin delivery method and/or glucose monitoring method within the last 3 months, of which they are unfamiliar<br>• Recent pregnancy or lactation (within the last 6 months)

	• Planning to get pregnant within the next 12 months<br>• Self-identifies as current or recent smoker (within the last 6 months)<br>• Significant weight change (+/-10% body weight) within the last 3 months<br>• Previous weight loss surgery<br>• History of malignancy (other than non-melanoma)<br>• History of thalassemia or other haemoglobinopathy<br>• Pre-existing anaemia<br>• Recent blood transfusion (within the last 3 months)<br>• A family history of premature heart disease<br>• Previously diagnosed familial hypercholesterolaemia<br>• Previously diagnosed gastrointestinal disease, liver disease, chronic kidney disease (CKD; eGFR <60), respiratory disease, thyroid disease or cardiovascular disease<br>• Previously diagnosed with a clinical eating disorder (within the last 5 years)<br>• Previously diagnosed neurological disorder<br>• Existing patient of Mrs Amy Rush (Credentialled Diabetes Educator), Dr David Lim (General Practitioner), Dr Kevin Lee (Endocrinologist) or Ms Jessica Turton (Accredited Practising Dietitian)

3. RATIONALE / BACKGROUND

Type 1 diabetes (T1D) is an autoimmune condition characterised by pancreatic beta cell destruction and absolute insulin deficiency. Individuals living with T1D must inject insulin multiple times a day to survive and can experience serious daily challenges associated with manual regulation of blood glucose levels. Modern T1D management is primarily focused on reducing risk and burden of chronic disease, including cardiovascular diseases, since the discovery of insulin in 1921 has extended the lives of individuals affected [1]. Achieving a haemoglobin A1c (HbA1c) of ≤7.0% is therefore the primary management target in diabetes [1] as it is associated with reduced risk of developing chronic complications related to hyperglycaemia [2]. However, the high amounts of exogenous insulin commonly being used to achieve glycaemic control targets in people with T1D also has long-term health consequences, including hyperinsulinemia, which is associated with obesity, the metabolic syndrome and atherosclerosis [3, 4]. Ultimately, adjunctive therapies that achieve glycaemic control *and* minimise reliance on excessive insulin are needed to improve the health and wellbeing of individuals living with T1D.

The varying impact of dietary factors on blood glucose levels is well-known, yet there remains a lack of consensus surrounding the optimal dietary approaches to achieve glycaemic control in T1D. Standard treatment involves multiple daily injections of insulin, wherein the dose of meal-time insulin is calculated based on the estimated grams or portions of carbohydrate consumed. Leading health authorities, including the National Health & Medical Research Centre (NHMRC), have traditionally recommended a high-carbohydrate (HC) diet, contributing 45-65% total energy intake (TEI), although this recommendation has been inferred from National Dietary Guidelines for the general population who are free from diabetes [1]. Data from T1D registries across 19 countries in Australasia, Europe and North America (n = 324,501) reported that 84% of patients exhibited HbA1c above the desirable target range (>7.0%) [5] suggesting that the current dietary advice for T1D is achieving limited impact and alternative dietary approaches should be considered.

A separate body of evidence conducted in type 2 diabetes (T2D) shows that diets containing less than 45% TEI from carbohydrates are effective for improving glycaemic control and CVD risk. Multiple systematic reviews have demonstrated that, when compared to usual care HC diets, diets <45% TEI from carbohydrates achieve greater reductions in HbA1c and use of diabetes medications [6-8] in addition to more favourable changes in blood lipid profile, including greater increases in HDL-cholesterol and decreases in triglycerides [8]. Moreover, there is a reported dose-response effect such that HbA1c is lower with greater carbohydrate restriction [6, 7]. Low-carbohydrate (LC) diets are not clearly defined in the scientific literature but in a recent systematic review (conducted by members of this team), which is the largest systematic review of all LC diets for T2D management available to-date (n=41; including all interventional study designs), LC diet interventions were defined as diets containing ≤130 g/day or 26% TEI from carbohydrate [9]. One 2-year trial (conducted by members of this team) compared a LC diet (14% TEI as carbohydrate) with a traditional HC diet (53% TEI as carbohydrate) in individuals with T2D and achieved greater reductions in diurnal glycaemic variability and diabetes medications alongside improvements in multiple CVD risk markers [10]. This cumulative evidence has led to recent updates to clinical practice guidelines globally, with acceptance of LC diets as an effective therapeutic option for T2D management by national organisations, including Diabetes Australia, Diabetes UK and the American Diabetes Association [11-13].

In response to this, the interest in LC diets for T1D management has increased. Qualitative evidence has found that many patients with T1D self-restrict carbohydrates, reporting that large amounts of carbohydrates coupled with large doses of required insulin lead to

unpredictable blood glucose levels [14]. Another study reported that a lower intake of dietary carbohydrates was associated with lower HbA1c levels in outpatients with T1D [15]. Members of this research team recently published the first systematic review of diets <45% TEI from carbohydrates for T1D management and nine studies were included (RCTs=2; pre-post interventions=4; retrospective case-series analyses=2; case-report=1) [16]. Six studies reported a mean reduction in HbA1c with diets <45% TEI from carbohydrates, with three statistically significant reductions (P<0.05) [16]. The LC diet arms (≤26% TEI from carbohydrates) also resulted in a reduced need for exogenous insulin while maintaining (or improving) glycaemic control [16]. One small RCT of 10 adults reported a 33% reduction in the mean total daily insulin use (TDI) with a LC diet (50-75 g/day) compared to no change with a diet higher in carbohydrates (~204 g/day) [17]. Another small RCT of 14 adults with T1D showed similar results [18]. No study in our review reported any statistically significant change in the negative direction for any T1D management outcome with a LC diet [16]. However, due to the significant heterogeneity, small sample sizes and quality of included studies, an overall effect of diets LC diets on T1D management could not be determined and additional research is needed.

Preliminary evidence suggests that LC diets appear safe for use in T1D and may be effective for certain subgroups of this clinical population. However, the available body of research is limited and there is still a lack of consensus regarding the efficacy of LC diets for improving T1D management. Since there are a multiplicity of lifestyle factors impacting T1D management [19], and with consideration that all individuals have their own personal needs and preferences, an interventional study that uses participants as their own controls and investigates a patient-led dietary approach will be of important clinical relevance. Therefore, a within-patient interventional study investigating a LC diet with an adaptive carbohydrate prescription (25-75 g/day) will be undertaken in adults with T1D in an outpatient setting.

4. AIMS / OBJECTIVES / HYPOTHESES

The aim of this research is to assess the efficacy of a LC diet in adults with T1D. Our primary objective is to determine the effects of a LC diet (25-75 g/day) on clinical markers of T1D management including HbA1c, glycaemic variability, frequency of hypoglycaemia, TDI, and quality of life.

In adults with T1D, compared to a habitual diet that is higher in carbohydrates, a LC diet will result in:

- improved glycaemic control (lower HbA1c, reduced glycaemic variability, reduced frequency of hypoglycaemia)
- reduced TDI requirements (wherein the reduction is expected to come primarily from meal-time insulin doses)
- improved quality of life and satisfaction with diabetes management

5. PARTICIPATING SITES

This project will include both online (Telehealth) and in-person contact. Online visits will be conducted via Telehealth using Zoom video conferencing (https://zoom.us). Clinic visits will be conducted from a private clinic room at the Susan Wakil Health Building (The University of Sydney, Camperdown NSW 2006). There is opportunity for fully remote participation in this study, if required or preferred. In addition, this study provides opportunity for a member of participants' usual diabetes care team to be involved. Before entry into the study, participants will be required to nominate one member of their usual diabetes care team who is proficient in insulin management (i.e., an endocrinologist, a general practitioner or a diabetes educator). The nominated healthcare practitioner (HCP) will be made fully aware in writing of their patient's intention to participate in the study and details of the intervention. The HCP will be asked to confirm their ability and willingness to provide ongoing support (usual care) and medical oversight (specifically, management of insulin therapy) to their patient(s) throughout the duration of the intervention from their usual practice location or via Telehealth on an as-needed basis.

6. RESEARCH PLAN / STUDY DESIGN

6.1 Type of study

A 28-week single arm, within-participant controlled intervention study.

6.2 Population / sample size

The primary outcome is HbA1c. Based on a clinically relevant difference in HbA1c of 0.7% (absolute) with a standard deviation of 1.0 [17], to achieve 80% power with alpha <0.05, we require a sample size of n=16. Anticipating a 20% dropout rate, we plan to recruit 20 participants into the study.

However, as this study is part of a PhD project, we've only got time and resourcing to have a recruitment period of 5 months. The recruitment period will end at 5 months and the study will continue, irrespective of the number of participants recruited up until that point (Table 2).

6.3 Statistical analyses

Data will be analysed using the most up-to-date version of SPSS. Outcome results will be presented as means and standard deviations. One-Way Analysis of Variance (ANOVA) will be used to assess the within group differences across time for each outcome.

6.4 Recruitment and selection of participants (including informed consent)

Participants will be recruited via public advertisement, including posters/flyers displayed at the University of Sydney, diabetes centres, the research site and on social media. We will also reach out to individual endocrinologists, general practitioners and diabetes educators with information on the study and interested practitioners can opt to receive a pack of flyers and Participant Information Sheets (PIS) to distribute as desired. Individuals wishing to take part in the study will be required to make initial contact with the research team by calling or emailing Ms Turton (contact details provided on advertising materials) and/or completing an online survey via REDCap (hyperlink and QR code displayed on advertising materials). The survey will involve multiple screening questions to determine whether or not interested individuals are eligible to participate in the study. Survey responses will be assessed in the order they are received by one investigator (JT) against the pre-specified inclusion/exclusion criteria. Participants will be asked to provide valid evidence to confirm their T1D diagnosis, the duration of their diabetes, and their recent HbA1c result (e.g., hospital discharge summary, specialist letter, pathology report). Ineligible participants will be excluded and provided the reason(s) for exclusion via email or phone. Potentially eligible participants will receive a phone call from JT to clarify responses, provide additional screening information (e.g., habitual dietary intake, completed confirmation of support letter from their nominated HCP) and if deemed eligible, schedule their first clinic visit. At this point, participants will also be asked to provide contact details of their usual GP so they can be informed of their patient's decision to be involved in the study (Attachment 53). The first 20 eligible volunteers will enter the study (Figure 1).

6.4.1 Inclusion criteria

- Adults aged 18-70 yrs
- Residing within Australia
- Previously diagnosed T1D (confirmed with specialist letter/correspondence, diabetes duration ≥6 months, using multiple daily injections of insulin and/or insulin pump)
- Suboptimal glycaemic control (HbA1c >7.0%)

- BMI 18.5-39.9 kg/m^2
- Habitual dietary intake contains >150 g/day of total carbohydrates (assessed by a 24-hour diet recall collected by study dietitian)
- Independent free-living, able to understand study requirements, speak and understand fluent English, and both physically and cognitively able to provide their informed consent
- Willing and able to self-monitor blood glucose levels using both finger-prick and/or continuous/flash blood glucose monitoring devices on a daily basis
- Willing and able to learn and utilise dietary carbohydrate and protein counting skills
- Willing and able to titrate insulin dosages according to their dietary carbohydrate (+/- protein) intake in consultation with a member of their usual diabetes care team
- Owns and can competently use an iOS or Android mobile device that is able to receive text messages/calls and download free mobile applications from the *App Store* or *Google Play*
- Has access to and can competently use a computer, mobile or tablet device with internet connection for uploading data onto REDCap and for Zoom conferencing
- Be willing to visit an Australian Clinical Labs collection centre (https://www.clinicallabs.com.au/location/) on four separate occasions throughout the study

6.4.2 Exclusion criteria

- HbA1c ≤7.0%
- Underweight (BMI <18.5 kg/m^2) or class III obesity (BMI ≥40 kg/m^2)
- Habitual use of an automated insulin delivery system to control blood glucose levels
- Habitual adherence to a fixed insulin regimen such that they are not confident in titrating insulin dosages according to their dietary intake.
- Previously diagnosed hypo unawareness
- Non-English speaking or unable to understand English
- Previous medical diagnosis of cognitive impairment and/or mental illness (not including depression and anxiety)
- Using medication to manage depression and/or anxiety and medications have been increased within the last three months
- Current physical impairment or disability that limits individual capacity to communicate with researchers and/or use the mobile application *Easy Diet Diary* (e.g., blind/vision impaired/deaf/speech impaired)
- Habitual dietary intake contains ≤150 g/day of total carbohydrates
- Usual dietary intake strictly excludes animal-based protein (e.g., vegan diet)

- Undertaken major change(s) to their insulin delivery method and/or glucose monitoring method within the last three months, of which they are unfamiliar (e.g., switched from injections to insulin pump and has never used pump before, or from multiple daily finger pricks to CGM and has never used CGM before)
- Recent pregnancy or lactation (within the last six months)
- Planning to get pregnant within the next 12 months
- Self-identifies as current or recent smoker (within the last 6 months)
- Significant weight change (+/- 10% body weight) within the last three months
- Previous weight loss surgery
- History of malignancy (other than non-melanoma)
- History of thalassemia or other haemoglobinopathy
- Pre-existing anaemia
- Recent blood transfusion (within the last three months)
- A known family history of premature heart disease, including having a first-degree male relative (i.e., father or brother) or a first degree female relative (i.e., mother or sister) who had a heart attack, stroke or was diagnosed with cardiovascular disease before the ages of 55 or 65, respectively
- Previously diagnosed familial hypercholesterolaemia
- Previously diagnosed gastrointestinal disease (including inflammatory bowel disease [IBD]; not including irritable bowel syndrome [IBS] or coeliac disease) unless condition is treated and stable (e.g., no change in medications within the last 3 month and asymptomatic)
- Previously diagnosed liver disease (including liver cirrhosis; not including fatty liver), chronic kidney disease (CKD; eGFR <60), respiratory disease (not including stable treated asthma), thyroid disease (including goitre; not including stable treated hyper- or hypothyroidism) or cardiovascular disease (including coronary heart disease, heart failure, cardiomyopathy, congenital heart disease, peripheral vascular disease and stroke; not including hyperlipidaemia or hypertension)
- Previously diagnosed neurological disorder (including high-frequency episodic migraine [10-14 headache days per month], chronic migraine [≥15 headache days per month], epilepsy, dementia, multiple sclerosis and Parkinson’s disease; not including low frequency migraines [<10 headache days per month])
- Previously diagnosed with a clinical eating disorder (including anorexia nervosa, bulimia nervosa or binge eating disorder) (within the last five years)

- Existing patient of Mrs Amy Rush (Credentialled Diabetes Educator), Dr David Lim (Medical Doctor), Dr Kevin Lee (Endocrinologist) or Ms Jessica Turton (Accredited Practising Dietitian)

6.5 Control Period

The schedule of events is clearly outlined in Table 1 and Figure 1. During the first study visit (start of week 0), Ms Turton will explain the health information that needs to be collected by the participants across the following seven days. This will involve wearing a continuous or flash blood glucose monitoring device and recording insulin dosages and food intake. Participants will be provided with the necessary devices and resources required to collect this information and instructed on how to use them (e.g., continuous blood glucose monitor, insulin logbook, online diet tracking App, kitchen scales). Participants will also be provided with a pre-paid pathology request form (from iMedical, https://imedical.com.au). The study dietitian will help the participants locate their nearest Australian Clinical Labs pathology collection centre and participants will be instructed on when to visit this centre (Figure 1). Participants will be provided with a resource ('Preparing for your blood test'; Attachment 49) to instruct them on safely preparing for their blood test. After seven days (end of week 0), participants will attend their local Australian Clinical Labs collection centre after an overnight fast (10-12 hours). Any participant who has needed to treat hypoglycaemia (<4.0 mmol/L) within the fasting window of their blood test will be advised to contact Jessica (study dietitian) and organise their blood test on another day (ideally, the following day). A trained pathologist will take a set of blood samples using standard procedures and the samples will be analysed by Australian Clinical Labs. Participants will be instructed to have a meal and drink fluids immediately (<30 mins) after their blood has been drawn.

Further outcome data, including quality of life, diet satisfaction, BMI, waist circumference, resting blood pressure, and physical activity level will also be collected on the same day (or within a reasonable timeframe i.e., ≤3 days). Ms Turton will also obtain results from the continuous/flash blood glucose monitor, insulin logbook and online diet tracking App from participants at this time-point. Outcome measurements will be directly inputted into REDCap by Ms Turton.

After all baseline data (for the control period) has been collected, Ms Turton will provide participants with instructions for the next four weeks which is to maintain usual patterns of eating, exercise and T1D management (Attachment 24). However, participants will be required to test their blood glucose levels at least six times daily, in accordance with standard

diabetes management practices, using whatever method(s) they usually use to test glucose. The dietitian will provide instructions and written resources on blood glucose monitoring (Attachment 47), managing hypoglycaemia (Attachment 48) and managing sick days (Attachment 46) (Table 1). There will be a diet session (Diet Session 0) scheduled in after two weeks and some basic diet education on carbohydrate counting (Attachment 25) and reading food labels (Attachment 26) will be provided (Table 1). During the last week of the control period, all of the same health assessments described above will be repeated (Table 1 and Figure 1). On the last day of the control period (end of Week 4), participants will have their first diet session of the intervention period (Diet Session 1) with the study dietitian and the intervention period will commence.

6.6 Intervention Period

6.6.1 LC diet

The dietary prescription is based on previously reported approaches shown to be safe and effective in multiple studies of LC diet interventions conducted in individuals with T2D [9].

This study will investigate a LC diet with an adaptive carbohydrate prescription that starts at 50 g of digestible carbohydrate per day and is then adapted (increased or decreased) within a broader range of 25-75 g/day according to individual blood glucose levels and personal preference. Participants will also be encouraged to distribute carbohydrates evenly across the day such that total carbohydrates do not exceed 20 grams at single eating occasion. To help participants reach nutritional adequacy, they will be encouraged to consume at least one serve of protein foods (150 g portion) with each main meal and to have three main meals daily, with or without snacks (participant preference). Participants will be given a food list with suggested food sources of proteins, fats, and carbohydrates to assist with meal structuring (Attachment 30). The intake of whole foods will be emphasised (e.g., vegetables, fruits, nuts, seeds, whole grains, meat, fish, eggs, yoghurt, milk, butter, unrefined fats and oils, etc.), while the intake of refined and highly processed food products (e.g., pastries, frozen meals, refined cereals, ice-cream, fruit juice, muesli bars, etc.) will be discouraged. Sample 3-day meal plans will also be provided to participants as a guide. There will be no upper limit intake restrictions on recommended sources of proteins and fats.

Recommendations for alcohol intake will be a maximum of two standard drinks daily with at least three alcohol-free days each week.

Individual dietary strategies will be geared toward helping participants meet the Recommended Daily Intake (RDI) and Adequate Intakes (AI) for essential nutrients including water (https://www.nrv.gov.au/nutrients), except for sodium wherein the estimated requirements for all participants are expected to increase as a result of lower insulin levels with adherence to the dietary intervention [20]. The adjusted recommendations for sodium in this intervention will be 3000-6000 mg/day for individuals with and without hypertension [21] to prevent sodium deficiency. Participants will be recommended to include at least 1.5 teaspoons of iodine-rich salt daily, as this will also help them meet their daily requirements for iodine. The recommended amount of salt will be provided to participants in pouches, and they will be instructed to measure out 1.5 teaspoons daily and add to foods and drinks.

Diet sessions will follow the structure outlined in Table 1. Participants will receive dietary instruction, strategies, and education, including written resources developed by the study dietitian (adapted from relevant NDSS facts sheets available from: https://www.ndss.com.au/about-diabetes/resources/). Dietary education will incorporate information on carbohydrates, proteins and fats, including carbohydrate and protein counting (given the need to take protein into account when calculating meal-time insulin requirements with habitual carbohydrate restriction <100 g/day [22]). Participants will be encouraged to use the *Easy Diet Diary* mobile application (Xyris software) throughout the study to assist with accurate carbohydrate and protein counting for calculating insulin requirements, and as a means of helping them achieve their carbohydrate prescription.

There will be opportunity to change the carbohydrate prescription after four and eight weeks. At these diet sessions, participants will discuss their dietary intake, blood glucose levels and individual preferences with the study dietitian. The study dietitian will provide an assessment based on their clinical judgement and together, the patient and dietitian will make a decision on whether or not to increase or decrease the carbohydrate prescription. In all cases, the adapted carbohydrate prescription will be specific (to the gram) and remain within the range of 20-75 g/day. E.g., 1: If a participant is struggling to adhere to a carbohydrate prescription of 50 g/day and is actually consuming 90 g/day, then the dietitian and participant might decide a prescription of 75 g/day is more achievable. E.g., 2: If a participant is closely adhering

to a 50 g/day but their blood glucose levels are still consistently above target after meals, then the dietitian and participant might decide a prescription of 30 g/day is required for better blood glucose control. This adaptive, patient-led approach is designed to take individual variations in dietary preferences and insulin sensitivity into account.

6.6.2 Insulin management & self-monitoring of blood glucose

The purpose of this study is to investigate a LC dietary intervention. However, it is not possible to make major changes to the dietary management of T1D without considering the subsequent required change(s) to insulin management. As such, all participants and their nominated HCP will be provided with an information pack (developed by the study diabetes educator, dietitian, physician and endocrinologist) and adapted from relevant NDSS fact sheets available from: https://www.ndss.com.au/about-diabetes/resources/) at the beginning of the intervention period that contains suggested guidelines for insulin management on a LC diet. The information pack includes resources relating to general considerations for insulin management on a LC diet; blood glucose self-monitoring; managing hypoglycaemia; and managing sick days. The information pack will be provided to participants by the study dietitian during Diet Session 1, and participants will get access to pre-recorded videos of the study diabetes educator explaining the resources. The information will also be discussed during a brief Telehealth session (~30 min) with the study diabetes educator, which will be scheduled within the first two weeks of the intervention period (Table 1, Figure 1). The purpose of this session with the study diabetes educator is to clarify the information contained in the resources, provide participants with an opportunity to ask questions about insulin management on a LC diet and to reduce the burden placed on participants' nominated HCP. This will be the only scheduled session with the study diabetes educator and all participants will be given regular reminders to follow up with their nominated HCP for all matters relating to insulin management, including individualised advice on insulin titrations. The study dietitian, diabetes educator, physician and endocrinologist will also be available throughout the duration of the study to provide support via Telehealth (phone or Zoom) to the participants' nominated HCPs, if needed.

Participants will be required to perform usual care practice self-monitoring blood glucose readings before each meal and two hours after meals (measured using their own blood glucose monitoring device) for insulin calculations and adjustments, and to

review their dietary carbohydrate prescriptions with the dietitian. Blood glucose targets will be consistent with standard practice diabetes targets and are:

- 4-8 mmol/L when fasting and before meals
- 4-10 mmol/L two hours after starting meals

Participants will be encouraged to share their glucose data with the study dietitian, either via their glucose monitoring software online or by downloading their glucose data from their glucose-monitoring device (which stores the blood glucose data) and providing it to the study dietitian via email or Zoom conferencing for discussion/review. This data will be used for monitoring purposes only and will not be used for the formal outcome analysis.

6.6.3 Self-monitoring ketones

For safety purposes, participants will be required to self-monitor either urine or blood ketones regularly throughout the intervention period using their usual method(s) of measuring ketones. Participants will be required to measure their ketones at least twice per week, and more under certain circumstances (e.g., if feeling unwell) as per standard of care diabetes practices. To assist participants in doing this, the required amount of ketone test strips will be provided. A resource titled, 'Self-monitoring ketones' (Attachment 52) will be provided at the beginning of the intervention period to instruct participants on how and when to monitor for ketones, what level of ketones are and are not expected, and what to do if they are experiencing unexpected changes in blood or urine ketones. The resource will help participants understand that mild, controlled ketone readings are normal and expected with the LC dietary intervention. Nutritional ketosis is typically defined as a ketone reading within the range of 0.5–3.0 mmol/L [23] and should not be confused with DKA that is associated with a rapid increase in ketone levels typically 10 times the level observed with nutritional ketosis. Nevertheless, we are taking a conservative approach and advising participants to maintain ketones ≤0.6 mmol/L, as per usual standard diabetes practice. The resource, 'Self-monitoring ketones' (Attachment 52), includes guidelines on when to monitor for ketones, and what action to take if ketones are >0.6 mmol/L. In brief, participants will be advised to refer to the resource titled 'Managing sick days' (Attachment 43) for more information. In any case, participants will be instructed to immediately inform Ms Turton (study dietitian) of any instances where ketones are >0.6 mmol/L and the case will be flagged with the study endocrinologist. The overseeing endocrinologist will then make a decision to either (a) continue as is or under certain condition (e.g., need to monitor

ketones once daily); (b) refer them to their nominated HCP or GP for independent review; or (c) refer them to the study diabetes educator or study physician for review. If Dr Lee is unavailable, then Dr Lim will be consulted to review the case. All cases referred will be documented and used to assess safety of the intervention in the data analysis and reporting.

6.6.4 Other medications

Participants taking other medications that might require adjustment during the intervention period, such as oral anti-glycaemics and anti-hypertensives, will be assessed by the study physician via a Telehealth Medical Assessment prior to the commencement of the intervention period, who will develop an individual medication management plan (Attachment 9). The purpose of this plan is for the study physician to flag any potential medication adjustments with participants' usual HCP and usual GP (unless this is the same practitioner). The study physician may also recommend the participant do additional self-monitoring (e.g., measure blood pressure once daily) to help guide any medication adjustments that may be needed. It is then ultimately up to participants' usual GP to make the decision on what precise medication adjustments are required, if any, throughout the study. The participant will be asked to report any medication changes to the study dietitian at each fortnightly diet session throughout the study so they can be recorded. Any medication change will be flagged with the study physician and a medical review will be scheduled if necessary, or the study physician will contact participants' usual GP to discuss the change(s).

If consent is provided by the participant, GPs will be notified of their patient's involvement in the study upon recruitment of the participant (their patient) and will be provided with a letter outlining study information (Attachment 53). The involvement of participants' usual GPs is not expected to place a significant increased burden on these practitioners because it is their duty of care to review and adjust medications for their patients on a regular basis, particularly when diet change is undertaken. The expected adjustments include reductions in or changes to anti-hypertensive and anti-hyperglycaemic medications [24]. The study physician and study endocrinologist will be available to consult with participants' usual GPs, if needed. Participants will be encouraged to continue following up with their usual GP for general medical issues and enquiries.

6.6.5 Physical activity

All participants will be encouraged to maintain their usual physical activity level throughout the entire duration of the study.

6.6.6 Outcome measurements (post-intervention)

During the last week of the intervention period (week 16), participants will be required to have their outcomes measured seven days apart. All health outcome measurements will be collected following the same procedures outlined in the control period. See Table 1 and Figure 1 for a detailed schedule of what outcomes will be measured and when throughout the entire study.

6.7 Follow Up

The end of the intervention period will mark the end of the active education period. However, participants will be asked to schedule one final follow-up Telehealth session with Ms Turton after another 12 weeks for the purposes of collecting follow-up outcome data (Figure 1). The purpose of this part of the study is to assess what dietary approach participants choose to take after the LC diet intervention, and then as a consequence of this – what is the impact of their diet on their HbA1c, quality of life and diet satisfaction. There will be no scheduled contact with the study dietitian, study physician or study diabetes educator during the follow-up period. Participants will be advised to follow up with their usual diabetes care team for all matters, as they were doing prior to entering the study. It will be the sole decision of the participants as to whether or not they continue to follow the LC diet. Participants will be able to keep all the resources that were provided to them throughout the course of the study.

Participants will be sent a reminder email two weeks prior to the scheduled session which will include relevant instructions. The only outcome data being measured at follow-up is HbA1c (collected at Australian Clinical Labs using a pre-paid form from iMedical), dietary intake (collected by the study dietitian using a 24-hour diet recall), total daily insulin dose (collected by participants using a 24-hour insulin logbook), quality of life (collected by participants using the DQOL Brief Clinical Inventory) [25] and diet satisfaction (collected by participants using a validated questionnaire [26]. Participants will be provided with a link to a follow-up survey hosted on REDCap where they can securely record and upload their follow-up data.

6.8 Schedule of events

The schedule of events from first contact to follow-up (final contact) is displayed in Figure 1. Participants will be sent text message reminders by Ms Turton the day before action is required from them (e.g., attend pathology collection centre, telehealth diet sessions).

6.9 Withdrawal / termination

Withdrawal from the study will be possible at any time and only data collected about the withdrawing participant up until that point will be included in the study. Participants will be asked for their reason(s) for withdrawing but will be under no obligation to answer. The date of withdrawal and reason for withdrawing (if provided) will be recorded in REDCap. There will be no consequences for withdrawing from the study.

Although unlikely, serious safety concerns may lead the researchers to terminate individual participation in the study or terminate the study entirely. For example, if a participant were to suffer a serious adverse event that was directly attributed to the study intervention. With consideration that the nature of this study is to investigate the effect(s) of a dietary intervention with a high level of professional support, we expect the risk of any adverse events to be minimal.

6.10 Informed consent process

Potential participants will be independently responsible for contacting the researchers to make initial contact and express their interest to participate in the study (via email, phone or completion of survey on REDCap to freely provide contact details). All participants will be required to complete the online survey hosted on REDCap which includes a downloadable PDF of the PIS. All potentially eligible participants will be required to provide their informed written consent by completing the online survey and ticking a box to confirm they have read the PIS and fully understand the study details.

6.11 Participant reimbursement

There will be no reimbursements provided to participants for being involved in the study.

6.12 Continuation of therapy

At the end of the study, participants will be advised to continue following up with their usual general practitioner and HCP(s) for all matters. Participants will be able to keep all the

resources provided to them throughout the intervention period and may wish to continue following the dietary intervention under the support of their usual HCP(s). Although Ms Turton, Amy Rush, Dr Lim and Dr Lee offer consultations in private practice, participation in this study does not necessarily allow for participants to remain clients of Ms Turton, Mrs Rush, Dr Lim or Dr Lee when the study is finished. If, on completion of the study, participants wish to secure an appointment with Ms Turton, Mrs Rush, Dr Lim or Dr Lee, they will be responsible for organising this and paying any future consulting fees arising from these consultations.

6.13 Confidentiality and Privacy

Participant information will be stored securely and kept strictly confidential, except as required by law. There will be no sharing of identifiable data with anyone not listed on this protocol, except for the participants' nominated HCP, without prior written consent from the participant. Refer to section 10 for full details on our data management plan, which is heavily focused on protecting participants' confidentiality and privacy at all times.

7. ETHICAL CONSIDERATIONS

We will obtain ethics approval from the University of Sydney and will comply with all standards set by their human ethics committee.

7.1 Avoiding real or perceived coercion (recruitment)

Our method of recruitment, as outlined in section 6.4, is via anonymous flyers and posters (distributed using multiple platforms), with all prospective patients reaching out to researchers. Therefore, there is no risk of real or perceived coercion in the recruitment process.

7.2 Privacy & confidentiality in the dissemination of research results

The overall results of this research project will be disseminated via journal publication(s), conference presentations, educational seminars and a student thesis. Results will be primarily reported as means and variances; individuals will not be identifiable in any dissemination of research results. Where reference to individual data is appropriate/necessary, this will be reported as "one participant", etc. rather than using participant ID codes or any potentially identifying information.

7.3 Potential risks involved with participation in research

7.3.1 Discomfort or infection

There is a small risk of discomfort infection associated with finger-prick blood sampling, application of glucose monitoring devices, and venipuncture blood draws. In all cases, standard antiseptic protocols and procedures will be used to minimise the risk of physical harm. It should be noted that the expected physical harm from participating in this research is typical of standard T1D management. For example, regular finger pricks (for blood glucose and ketone testing) and insulin injections are required of all T1D patients, irrespective of their dietary management protocols. It is acknowledged that some patients may not be familiar with the use of a continuous or flash blood glucose monitoring (CGM or FGM) device, and in these cases, the study dietitian will take additional time to explain the risks/benefits and expectations of applying and wearing this device. The devices used are commercially available and will be used for their proposed purpose (https://eshop.medtronic-diabetes.com.au/en/cgmsupplies/sensors; https://www.freestylelibre.com.au/freestyle-libre-au-sensor.html).

7.3.2 Hypoglycaemia

Hypoglycaemia is a common acute complication of T1D which generally results from the difficulties in predicting expected rises in blood glucoses levels and accurately calculating insulin requirements. Due to the expected change(s) in insulin requirements as a result of the diet intervention, there is a risk of participants miscalculating their insulin requirements in the early stage of the intervention which could lead to increased frequency and/or severity of hypoglycemia. This will be mitigated by close monitoring of blood glucose levels, regular contact with the study dietitian, ongoing support from a nominated HCP, and oversight/supervision by the study physician, study diabetes educator and study endocrinologist.

Hypoglycaemia is defined by Diabetes Australia as a blood glucose level below 4 mmol/L [27]. The resources developed by the study dietitian and diabetes educator titled ‘Managing hypoglycaemia’ (Attachments 42 and 48) provide participants with information and guidelines on preventing and/or managing hypoglycaemia during the study. Participants will be required to test their blood glucose levels at least six times every day (i.e., before and 2 hours after each meal) using their own glucose monitoring

device, as per standard of care diabetes practice. They will also be encouraged to test their blood glucose levels before going to bed and before driving or participating in exercise. Participants will also be advised to test their glucose more often if they are: feeling sick or stressed; more or less physically active; experiencing more hypos than usual; experiencing changes in their routine; experiencing night sweats or morning headaches; noticing frequent instances of high blood glucose levels; and/or starting on new medications. Participants will be asked to provide their blood glucose data from their blood glucose monitoring device to the dietitian for review every fortnight throughout the study. If using a CGM or flash glucose monitor (FGM), any reading(s) <4 mmol/L should be confirmed with a finger prick device. If two or more readings across a 14-day period are <4.0 mmol/L then the dietitian will flag the case with the study endocrinologist. The overseeing endocrinologist will then make a decision to either (a) continue until the next review (14 days); (b) refer them to their nominated HCP or GP for independent review; or (c) refer them to the study diabetes educator or study physician for review. If Dr Lee is unavailable, then Dr Lim will be consulted to review the case. All cases referred will be documented and used to assess safety of the intervention in the data analysis and reporting. Participants with hypo unawareness will be excluded from participating in the study. However, if any participant believes they might have developed hypo unawareness during the study, they will be advised to contact Jessica Turton (study dietitian) as soon as possible, who will flag the case with the study endocrinologist to be assessed on an individual basis.

7.3.3 Dehydration or sodium deficiency (including hyponatraemia)

Due to the expected lowering of insulin requirements and circulating blood insulin in participants adhering to the LC diet, there is a risk of sodium deficiency or dehydration given insulin's role in sodium retention and sodium's role in fluid retention. Hyponatraemia is defined as a serum sodium concentration <135 mEq/L [28]. Although sodium requirements are increased on a LC diet, the diet is also expected to improve blood glucose control, and given that hyperglycaemia is a cause of hyponatraemia in individuals with diabetes [29], we expect the absolute risk of hyponatraemia to be low. Nevertheless, a resource titled, 'Importance of Sodium & Hydration' (Attachment 51) will be provided to participants at the beginning of the intervention period to provide specific instructions on minimising the risk of developing hyponatraemia and/or dehydration. To mitigate this risk, sodium recommendations will be increased relative to the Suggested Dietary Target (2000 mg/day) to 3000-6000 mg/day. Participants will

also be provided with the recommend amount of salt (provided as sealed salt pouches) and instructed to measure out 1.5 tsp and add to food and drink over the course of each day. In addition, minimally processed foods that contain sodium, such as cheese, olives, pickles and canned fish, will be recommended.

Participants will also be monitored throughout the study. Serum sodium levels will be measured at three time-points throughout the study (1. pre-control period, 2. post-control period/pre-intervention period, 3. post-intervention period) (Section 9.3). If a participant is detected to have mild hyponatraemia (serum sodium concentration 130-134 mEq/L [30]) measured at any time point throughout the study, the study physician will provide an immediate assessment of the patient (via Telehealth) and advise the patient and study dietitian on what steps need to be taken (e.g., increasing sodium intake, reviewing medications, etc.). In cases of moderate or severe hyponatraemia (<130 mEq/L) [30]), the participant will be contacted immediately and advised to seek emergency medical treatment. Symptoms that may indicate a greater need for salt will be monitored by the study dietitian at each diet session (once a fortnight) and include cold extremities, dark urine, dry mouth, postural dizziness, salt cravings, thirst, muscle cramps, constipation, light-headedness, low energy and headaches. If a participant reports one or more symptoms as being “moderate” or “severe” in intensity or frequency, the dietitian will advise them to increase their daily intake of salt by half a teaspoon and encourage them to drink water to thirst. A similar approach was used in a larger clinical trial investigating ketogenic diets (<30 g/day) in patients with type 2 diabetes (n=262) [23]. If a participant reports one or more symptoms as being “severe” in intensity or frequency, the dietitian will also flag the case with the study physician and a medical assessment will be organised for the participant if deemed necessary.

7.3.4 Diabetic ketoacidosis (DKA)

The National Evidence-Based Clinical Care Guidelines for Type 1 Diabetes in Children, Adolescents and Adults (2011) [1] states “DKA is life-threatening acute complication of type 1 diabetes. It occurs when insulin delivery is insufficient to prevent progressive hyperglycaemia and ketone body formation. Like hypoglycaemia, DKA can occur in anyone with type 1 diabetes, but is more common when there is a precipitant (e.g., infection of the gastrointestinal, respiratory or urinary tract). Aiding self-care, adherence to therapy and ‘sick day’ management can prevent many cases

of threatened or mild DKA from worsening and requiring hospitalisation." DKA is a medical emergency that should only be treated in hospital. Steps taken in this study are aimed at *preventing* the development of DKA in participants. If any participant at any time-point throughout the study is experiencing symptoms of ketoacidosis, they should dial 000 or present to emergency immediately.

Given that participants in this study must have been diagnosed with T1D and receiving insulin therapy for at least six months, the risk of any participant developing DKA would most likely be associated with inappropriate sick day management or cessation of insulin therapy. To mitigate the risk of DKA, participants will receive ongoing support from a member of their usual diabetes team (an endocrinologist, a diabetes educator or a general practitioner) for all matters related to insulin therapy and general diabetes management, including the prevention of DKA. In addition, participants and their nominated HCP will receive an information pack at the start of the study which will include the resources, 'Managing sick days' (Attachments 43 and 46), which provide information on DKA and how to monitor for and prevent DKA when participants are feeling unwell.

For safety purposes, participants will be required to self-monitor either urine or blood ketones regularly throughout the intervention period using their usual method(s) of measuring ketones. Participants will be required to measure their ketones at least twice per week, and more under certain circumstances (e.g., if feeling unwell) as per standard of care diabetes practices. To assist participants in doing this, the required amount of ketone test strips will be provided. A resource titled, 'Self-monitoring ketones' (Attachment 52) will be provided at the beginning of the intervention period to instruct participants on how and when to monitor for ketones, what level of ketones are and are not expected, and what to do if they are experiencing unexpected changes in blood or urine ketones. The resource will help participants understand that mild, controlled ketone readings are normal and expected with the LC dietary intervention. Nutritional ketosis is typically defined as a ketone reading within the range of 0.5–3.0 mmol/L [23] and should not be confused with DKA that is associated with a rapid increase in ketone levels typically 10 times the level observed with nutritional ketosis. Nevertheless, we are taking a conservative approach and advising participants to maintain ketones ≤0.6 mmol/L, as per usual standard diabetes practice. The resource, 'Self-monitoring ketones' (Attachment 52), includes guidelines on when to monitor for ketones, and what action to take if ketones are >0.6 mmol/L. In brief, participants will

be advised to refer to the resource titled 'Managing sick days' (Attachment 43) for more information. In any case, participants will be instructed to immediately inform Investigator Turton (study dietitian) of any instances where ketones are >0.6 mmol/L and the case will be flagged with the study endocrinologist. The overseeing endocrinologist will then make a decision to either (a) continue as is or under certain condition (e.g., need to monitor ketones once daily); (b) refer them to their nominated HCP or GP for independent review; or (c) refer them to the study diabetes educator or study physician for review. If Dr Lee is unavailable, then Dr Lim will be consulted to review the case. All cases referred will be documented and used to assess safety of the intervention in the data analysis and reporting.

7.3.5 Psychological or emotional stress

Any change(s) in chronic disease management, including an increased awareness of disease state (e.g., increased frequency of blood glucose monitoring), can be psychologically demanding and/or emotionally distressing. Health assessments included as part of the study may also bring up underlying psychological/emotional issues (e.g., emotional relationships with food). At the onset of the study (start of control period, Table 1), participants will be provided with an NDSS fact sheet relating to diabetes distress which includes contact details for free counselling/support services that participants can access at any time during or after the study (https://www.ndss.com.au/wp-content/uploads/fact-sheets/fact-sheet-diabetes-distress.pdf.). Participants will also be provided opportunity to discuss any issues they may be experiencing with the study dietitian during regular diet consults. The dietitian will flag any psychological issues or emotional stress with the study physician or the participants' nominated HCP and/or GP who may then refer participants to the appropriate health professional service for management.

7.3.6 Social distress

There is also a risk of social distress with increased travel and time commitments associated with participation in the study given participants will need to attend several study visits, diet sessions and pathology collection centres. To reduce the burden on participants, diet sessions 2-5 have been organised as Telehealth consults (via Zoom video conference) and there is opportunity to complete all study sessions remotely, if required or preferred. Sessions will be scheduled at a time of day that is most suitable

for the participants (including weekends and outside of working hours if necessary, to not affect participants' usual routines).

8. SAFETY CONSIDERATIONS

8.1 Adverse event definitions

For the purposes of this study, we will define adverse events as any incident that results in the participant requiring health care of which is directly attributed to involvement in the study. The expected risk of serious adverse events experienced as a result of the dietary intervention is low. However, participants will be advised to report details of any adverse events directly to the study dietitian or physician at any point during the control or intervention periods. In the unexpected case of a medical emergency, participants will be encouraged to dial 000 immediately and follow-up with researchers after medical treatment/assessment has been received and their condition is stable.

Adverse events have been described in detail in section 7.3 as potential risks that may be involved with participation in this research and include;

- Discomfort or infection(s) at the site of CGM/FGM, finger prick or venepuncture
- Increased frequency of hypoglycaemia
- Dehydration or sodium deficiency (e.g., headaches, muscle cramps, low energy, digestive discomfort)
- Diabetic ketoacidosis
- Psychological/emotional distress
- Social distress

8.2 Assessment and documentation of adverse events

We will use the University of Sydney's non-clinical trials adverse events reporting form as this is the most appropriate form for our study. All adverse events will be documented by Ms Turton as soon as notification about the event is received. Ms Turton will notify the study physician immediately who will assess the situation and make a decision as to what action is required to prevent recurrence of the event (e.g., changing the design of the study, stopping the study, making changes to the participant information sheet, etc.).

9. OUTCOMES

All primary, secondary and additional outcomes will be measured at three time-points: (1) pre-control period, (2) post-control period / pre-intervention period, and (3) post-intervention period. The only data being collected at follow up (12 weeks post-intervention) is HbA1c, total daily insulin (previous 24 hours), dietary intake (previous 24 hours), diabetes-related quality of life and diet satisfaction. Refer to Table 1 for a clear description of what outcomes are being measured at what time-points throughout the duration of the study for each participant. Refer to Figure 1 for an overview of this information.

9.1 Primary outcome

- Haemoglobin A1c, HbA1c (% and mmol/mol)*

9.2 Secondary outcomes

- Glycaemic variability (GV) markers (including Standard Deviation (SD) intraday and mean amplitude of glycaemic excursions (MAGE) [derived from 7-day CGM/FGM data]
- Frequency of hypoglycaemia (<3.5 mmol/L with or without symptoms) [derived from 7-day CGM/FGM data]
- Total daily insulin (TDI) [the sum of all long-acting and rapid-acting insulin given over a 24-hour period derived from a 3-day self-report insulin log]
- Quality of life [measured using the 15-item DQOL Brief Clinical Inventory [25]]

GV markers have been identified as independent risk factors for diabetes complications including CVD, underscoring the importance of this assessment to more rigorously evaluate the effect(s) of the diet intervention on diabetes management.

9.3 Additional outcomes

- Body Mass Index (BMI) (kg/m^2) [height and weight measured using standard procedures by study dietitian^]
- Waist circumference (cm) [measured at the midline point between the lowest rib and iliac crest by study dietitian^]
- Resting blood pressure (BP) (mmHg) [measured after 5 minutes of seated rest by study dietitian^]
- Fasting blood glucose (mmol/L)*

- Kidney function (Sodium, Potassium, Chloride, Bicarbonate, Urea, Creatinine, eGFR, Calcium, Corrected Calcium, Phosphate, Uric Acid)*
- Liver function (Total Protein, Albumin, Alkaline Phosphatase, Total Bilirubin, Gamma GT, AST, ALT, Globulin, Magnesium, Creatine Kinase)*
- Lipid studies (Cholesterol, HDL/LDL, Non-HDL Cholesterol, Triglycerides, LDL/HDL Ratio, Chol/HDL Ratio)*
- Physical activity level [self-report using the International Physical Activity Questionnaire [31]]
- Dietary intake (total energy, digestible carbohydrate, dietary fibre, protein, total fat, saturated fat, monounsaturated fat, polyunsaturated fat as kJ/day and kcal/day or g/day) [derived from self-report 3-day weighed food record collected using Xyris *Easy Diet Diary* and analysed using Xyris *FoodWorks Professional Edition*]
- Diet satisfaction [self-report using a 6-item questionnaire [26]]

*All biological samples will be collected and analysed by a NATA-Accredited Laboratory (Australian Clinical Labs) using standard procedures.

^Participants completing their clinic visits remotely will be asked to visit their nominated healthcare practitioner or local pharmacy to have anthropometric outcomes measured and recorded. Written instructions will be provided.

10. DATA MANAGEMENT

10.1 Data collection

We will be collecting data from the following sources:

- Online survey (including consent form)
- Email correspondence (participants may email data to Ms Turton)
- Clinical documents provided by participants and/or their HCPs
- Pathology results provided by iMedical
- Outcome measurements taken at study visits with the study dietitian (e.g., weight, waist circumference, blood pressure, questionnaires)
- Self-monitoring data provided by participants (e.g., glucose logs, insulin dosing logs, dietary intake data)

- Any other relevant information provided by participants at any time-point throughout the study (e.g., details of adverse events, change(s) to other medications, etc.)

All data collected about participants for this study will be promptly recorded or uploaded directly onto the University's preferred platform of REDCap. REDCap is a secure, password-protected online database platform, with access to this project limited to the individuals named on this ethics application. REDCap allows participants to upload relevant documentation that may be required during the online screening survey (e.g., evidence of T1D diagnosis). If participants do not have the relevant documentation available at the time of completing the survey, they may email it to Ms Turton or provide it in-person once obtained. In these cases, Ms Turton will be responsible for uploading the data collected via email (electronic document/s) or in-person (hard document/s) onto REDCap as soon as possible, before destroying the original electronic document/s (from her email inbox and/or downloads folder) or returning the hard document/s to participants. Data collected by the study dietitian or study physician at any time-point throughout the study will be recorded directly into specific data collection templates hosted on REDCap.

10.2 Data storage

As mentioned in section 10.1 all participant data collected during this study will be stored securely on REDCap. Data stored on REDCap will include documents containing personal identifiers and consent information. However, when data is exported from REDCap or extracted manually by researches for data analyses, only participant ID codes will be used and no data containing personal identifiers will be stored in any location other than securely on REDCap. Microsoft Word and Microsoft Excel will be used for merging and analysing data for this study (data synthesis and analysis). These files will be stored in a designated password-protected folder on Dropbox (study folder) and accessible by members of this research team using their individual account details and passwords.

<u>Original electronic documents</u> (containing participant identifiers) will not be saved in any form except on REDCap, in which case it will remain stored on REDCap securely until the end of the study. Any data that has been downloaded onto a computer or laptop will be deleted from the default downloads folder. Any data that has been received via email will be deleted from the email inbox and deleted folders.

Original hard copy documents that might be provided by the participants during in-person clinic visits will be scanned and stored securely on REDCap. Hard copy documents will be immediately shredded or returned to the participant (only if provided by the participant).

The master code sheet that links participant details with their IDs will be stored, separately to all other data, on the C:Drive of Ms Turton's password protected laptop. The master code sheet will be manually backed up to an encrypted and password-protected external hard drive once a month.

After the project has been completed, the master code sheet will be deleted such that the data will no longer be re-identifiable and it will not be possible to trace any study information back to individuals participants. The external hard drive containing the back-up code sheets will also be adequately destroyed by the research team (all data will be deleted and then the hard drive will be reformatted to prevent any 'memory' of the data). Consent information will be exported from REDCap and stored in the Sydney University repository for five years (as per HREC requirements) following the completion of data collection. Full deletion of data from the REDCap database will occur after the project has closed (after the final participant has completed data collection, and all required data have been adequately stored). A copy of the study folder (de-identified data only) will be made and stored on an open access data sharing platform such as the Open Science Framework (OSF) (https://osf.io/) in order to allow other researchers access to the raw data for their own projects (e.g., systematic reviews), if necessary.

10.3 Data usage

Relevant data items will be extracted or exported from REDCap in re-identifiable format (using study IDs only) and inputted into Microsoft Excel where all relevant data collected for each participant throughout the duration of the study will be merged for analysis. Data will be manipulated/analysed by study investigators on password-protected laptops or computers during the project.

11. TIMELINES / MILESTONES

Study milestones are outlined in Table 2. We anticipate the project to start on 21st June 2021 and end on 20th June 2022 (12 months). The recruitment period will start on 21st June 2021

and end on the 30th November 2021 (~5 months) or when 20 participants have been successfully recruited (whichever one comes first). Participants will enter the study at different time points, depending on when they are recruited. Including the follow-up period, the total time each participant will be in the study is 28 weeks (4-week control period, 12-week intervention period, 12-week follow-up period) or seven months. The primary data analysis will occur once the last participant has completed the 12-week intervention period (~18 weeks after making first contact with researchers and completing REDCap pre-screening survey), which is anticipated to be on or before 31st March 2022 (if the last participant is recruited on 30th November 2021). The last point of data collection will be completed on or before 30th June 2022 (end of the last participant's follow-up period) and the preceding time will be used to analyse the follow-up data, complete report writing, complete data management (store data securely according to data management plan) and finish the project.

12. FINANCIAL

This study has been partially funded by The Commonwealth Scientific and Industrial Research Organisation (CSIRO) who have kindly agreed to donate $6000.00 to this research project. Ms Turton also plans to raise $3000.000 through crowd sourcing activities. The anticipated cost of the study is $8211.20 (Attachment 18). If any unexpected costs arise throughout the study, additional support may be provided by the host institution via the Postgraduate Research Support Scheme (PRSS).

Ms Turton will undertake the role of the essential study dietitian, in addition to a project management role, as the study will form a major part of her PhD thesis. Ms Turton has been awarded a Research Training Program (RTP) Stipend.

It is confirmed that in-kind support will be provided by the Type 1 Diabetes Family Centre to cover the costs associated with Mrs Amy Rush's time spent on the project as the study diabetes educator.

Dr Lim and Dr Lee will provide in-kind time to perform the roles of the study physician and study endocrinologist, respectively.

13. PUBLICATION POLICY / DISSEMINATION OF RESULTS

As discussed in section 7.2, the overall results of this research project will be disseminated via journal publication(s), conference presentations, educational seminars and a student

thesis. Ms Turton will be primarily responsible for taking the lead in publication given the project forms a major part of her PhD thesis. All researchers will be acknowledged in publications relating to this project.

14. REFERENCES

[1] Craig M, Twigg S, Donaghue K, Cheung N, Cameron F, Conn J, et al. National evidence-based clinical care guidelines for type 1 diabetes in children, adolescents and adults. Canberra: Australian Government Department of Health and Ageing; 2011.
[2] Cavero-Redondo I, Peleteiro B, Álvarez-Bueno C, Rodriguez-Artalejo F, Martínez-Vizcaíno V. Glycated haemoglobin A1c as a risk factor of cardiovascular outcomes and all-cause mortality in diabetic and non-diabetic populations: a systematic review and meta-analysis. BMJ Open. 2017;7:e015949.
[3] Kelly CT, Mansoor J, Dohm GL, Chapman WH, 3rd, Pender JRt, Pories WJ. Hyperinsulinemic syndrome: the metabolic syndrome is broader than you think. Surgery. 2014;156:405-11.
[4] Wang MY, Yu X, Lee Y, McCorkle SK, Clark GO, Strowig S, et al. Iatrogenic hyperinsulinemia in type 1 diabetes: its effect on atherogenic risk markers. Journal of diabetes and its complications. 2013;27:70-4.
[5] McKnight JA, Wild SH, Lamb MJ, Cooper MN, Jones TW, Davis EA, et al. Glycaemic control of Type 1 diabetes in clinical practice early in the 21st century: an international comparison. Diabetic medicine : a journal of the British Diabetic Association. 2015;32:1036-50.
[6] Sainsbury E, Kizirian NV, Partridge SR, Gill T, Colagiuri S, Gibson AA. Effect of dietary carbohydrate restriction on glycemic control in adults with diabetes: A systematic review and meta-analysis. Diabetes research and clinical practice. 2018;139:239-52.
[7] Snorgaard O, Poulsen GM, Andersen HK, Astrup A. Systematic review and meta-analysis of dietary carbohydrate restriction in patients with type 2 diabetes. BMJ open diabetes research & care. 2017;5:e000354.
[8] Huntriss R, Campbell M, Bedwell C. The interpretation and effect of a low-carbohydrate diet in the management of type 2 diabetes: a systematic review and meta-analysis of randomised controlled trials. European journal of clinical nutrition. 2018;72:311-25.
[9] Turton J, Brinkworth GD, Field R, Parker H, Rooney K. An evidence-based approach to developing low-carbohydrate diets for type 2 diabetes management: A systematic review of interventions and methods. Diabetes, Obesity and Metabolism. 2019;21:2513-25.

[10] Tay J, Thompson CH, Luscombe-Marsh ND, Wycherley TP, Noakes M, Buckley JD, et al. Effects of an energy-restricted low-carbohydrate, high unsaturated fat/low saturated fat diet versus a high-carbohydrate, low-fat diet in type 2 diabetes: A 2-year randomized clinical trial. Diabetes, obesity & metabolism. 2018;20:858-71.
[11] Diabetes Australia. Position Statement: Low carbohydrate eating for people with diabetes. Diabetes Australia; 2018.
[12] Diabetes UK. Position statement: Low-carb diets for people with diabetes 2017.
[13] Davies MJ, D'Alessio DA, Fradkin J, Kernan WN, Mathieu C, Mingrone G, et al. Management of Hyperglycemia in Type 2 Diabetes, 2018. A Consensus Report by the American Diabetes Association (ADA) and the European Association for the Study of Diabetes (EASD). Diabetes Care. 2018;41:2669-701.
[14] Lawton J, Rankin D, Cooke DD, Clark M, Elliot J, Heller S. Dose Adjustment for Normal Eating: a qualitative longitudinal exploration of the food and eating practices of type 1 diabetes patients converted to flexible intensive insulin therapy in the UK. Diabetes research and clinical practice. 2011;91:87-93.
[15] Buyken AE, Toeller M, Heitkamp G, Irsigler K, Holler C, Santeusanio F, et al. Carbohydrate sources and glycaemic control in Type 1 diabetes mellitus. EURODIAB IDDM Complications Study Group. Diabetic medicine : a journal of the British Diabetic Association. 2000;17:351-9.
[16] Turton JL, Raab R, Rooney KB. Low-carbohydrate diets for type 1 diabetes mellitus: A systematic review. PLOS ONE. 2018;13:e0194987.
[17] Krebs JD, Parry Strong A, Cresswell P, Reynolds AN, Hanna A, Haeusler S. A randomised trial of the feasibility of a low carbohydrate diet vs standard carbohydrate counting in adults with type 1 diabetes taking body weight into account. Asia Pacific journal of clinical nutrition. 2016;25:78-84.
[18] Schmidt S, Christensen MB, Serifovski N, Damm-Frydenberg C, Jensen JB, Floyel T, et al. Low versus high carbohydrate diet in type 1 diabetes: A 12-week randomized open-label crossover study. Diabetes, obesity & metabolism. 2019;21:1680-8.
[19] Piłaciński S, Zozulińska-Ziółkiewicz DA. Influence of lifestyle on the course of type 1 diabetes mellitus. Arch Med Sci. 2014;10:124-34.
[20] Tiwari S, Riazi S, Ecelbarger CA. Insulin's impact on renal sodium transport and blood pressure in health, obesity, and diabetes. American Journal of Physiology-Renal Physiology. 2007;293:F974-F84.
[21] Mente A, O'Donnell M, Rangarajan S, Dagenais G, Lear S, McQueen M, et al. Associations of urinary sodium excretion with cardiovascular events in individuals with and without hypertension: a pooled analysis of data from four studies. Lancet (London, England). 2016;388:465-75.

[22] Krebs JD, Arahill J, Cresswell P, Weatherall M, Parry-Strong A. The effect of additional mealtime insulin bolus using an insulin-to-protein ratio compared to usual carbohydrate counting on postprandial glucose in those with type 1 diabetes who usually follow a carbohydrate-restricted diet: A randomized cross-over trial. Diabetes, obesity & metabolism. 2018;20:2486-9.
[23] Hallberg SJ, McKenzie AL, Williams PT, Bhanpuri NH, Peters AL, Campbell WW, et al. Effectiveness and Safety of a Novel Care Model for the Management of Type 2 Diabetes at 1 Year: An Open-Label, Non-Randomized, Controlled Study. Diabetes Therapy. 2018;9:583-612.
[24] Murdoch C, Unwin D, Cavan D, Cucuzzella M, Patel M. Adapting diabetes medication for low carbohydrate management of type 2 diabetes: a practical guide. Br J Gen Pract. 2019;69:360-1.
[25] Burroughs TE, Desikan R, Waterman BM, Gilin D, McGill J. Development and Validation of the Diabetes Quality of Life Brief Clinical Inventory. Diabetes Spectrum. 2004;17:41-9.
[26] Wolever TMS, Chiasson J-L, Josse RG, Leiter LA, Maheux P, Rabasa-Lhoret R, et al. Effects of Changing the Amount and Source of Dietary Carbohydrates on Symptoms and Dietary Satisfaction Over a 1-Year Period in Subjects with Type 2 Diabetes: Canadian Trial of Carbohydrates in Diabetes (CCD). Canadian Journal of Diabetes. 2017;41:164-76.
[27] Diabetes Australia. Hypoglycaemia. 2020.
[28] Verbalis JG, Goldsmith SR, Greenberg A, Korzelius C, Schrier RW, Sterns RH, et al. Diagnosis, evaluation, and treatment of hyponatremia: expert panel recommendations. The American journal of medicine. 2013;126:S1-42.
[29] Liamis G, Tsimihodimos V, Elisaf M. Hyponatremia in Diabetes Mellitus: Clues to Diagnosis and Treatment. Journal of Diabetes and Metabolism. 2015;6.
[30] Waikar SS, Mount DB, Curhan GC. Mortality after hospitalization with mild, moderate, and severe hyponatremia. The American journal of medicine. 2009;122:857-65.
[31] Craig CL, Marshall AL, Sjostrom M, Bauman AE, Booth ML, Ainsworth BE, et al. International physical activity questionnaire: 12-country reliability and validity. Medicine and science in sports and exercise. 2003;35:1381-95.

15. APPENDICES

Table 1. Outline of clinic visits and diet sessions throughout the study

Control Period	
Visit/session	*Outline*
**Clinic visit 1**<br>(start of week 0)	• Study schedule explained (Figure 1) and all visits/sessions for the control and intervention periods scheduled (inc. all sessions with study dietitian and study diabetes educator).<br>• Medical assessment scheduled, if necessary (i.e., for patients taking any medication(s) other than insulin).<br>• Equipment, demonstration and instructions provided for:<br>- CGM/FGM device (sensor(s) and reader) (7-day monitoring)<br>- Insulin logbook (3-day monitoring)<br>- Diet record (participants will be shown how to download and use the Easy Diet Diary mobile app to accurately record their dietary intake) (3-day monitoring)<br>- Biological samples (Australian Clinical Labs request form provided, and nearest pathology collection centre to the participant identified)<br>• Control period educational resources discussed:<br>o general recommendations<br>o NDSS fact sheet: Diabetes distress<br>o preparing for your blood test<br>• Participants encouraged to report any adverse events throughout the study.<br>• Participants encouraged to maintain usual patterns of eating, activity and diabetes management.
**Clinic visit 2**<br>(end of week 0)	• *Morning of clinic visit 2:* Participants attend their nearest Australian Clinical Labs collection centre (fasted) to have biological samples taken. Participants instructed to have a meal and drink fluids after blood draw and before their clinic visit.<br>• Data collected (pre-control outcomes):<br>- Return/upload CGM/FGM device/data<br>- Return/upload insulin logbook<br>- Return/upload diet record<br>- Anthropometric measurements taken (weight, height, waist circumference and blood pressure)<br>- Questionnaires completed (quality of life, physical activity and diet satisfaction)<br>• Participants encouraged to maintain usual patterns of eating, activity and diabetes management.<br>• Control period educational resources discussed:<br>o blood glucose monitoring<br>o managing hypoglycaemia<br>o managing sick days
**Diet Session 0**<br>(during week 2)	• Telehealth call (Zoom) with study dietitian.<br>• Participants encouraged to maintain usual patterns of eating, activity and diabetes management.<br>• Control period educational resources provided:<br>- carbohydrate counting<br>- reading food labels
**Medical Assessment**	• Telehealth call (Zoom) with study physician (if required).<br>• Medical assessment completed.

(during week 3 or 4)	• Medication management plan completed (copy provided to participant, their nominated HCP and their usual general practitioner).
**Clinic visit 3** (start of week 4)	• Equipment, demonstration and instructions provided for:<br>- CGM/FGM device (sensor(s) and reader) (7-day monitoring)<br>- Insulin logbook (3-day monitoring)<br>- Diet record (Easy Diet Diary mobile app) (3-day monitoring)<br>- Biological samples (Australian Clinical Labs request form provided)<br>• Participants encouraged to maintain usual patterns of eating, activity and diabetes management.
**Clinic visit 4** (end of week 4)	• *Morning of clinic visit 4:* Participants attend their nearest Australian Clinical Labs collection centre (fasted) to have biological samples taken. Participants instructed to have a meal and drink fluids after blood draw and before their clinic visit.<br>• Data collected (post-control/pre-intervention outcomes):<br>- Return/upload CGM/FGM device/data<br>- Return/upload insulin logbook<br>- Return/upload diet record<br>- Anthropometric measurements taken (weight, height, waist circumference and blood pressure)<br>- Questionnaires completed (quality of life, physical activity, diet satisfaction)<br>• Participants encouraged to have a short break prior to diet session 1.
Intervention Period	
Visit/session	*Outline*
**Diet session 1** (end of week 4) *Note – same day as clinic visit 4*	• Brief rationale for dietary intervention explained by study dietitian.<br>• Initial carbohydrate target set, and meal plan provided (50 g/day).<br>• Hardcopy diet resource book provided (all LC diet resources except meal plans).<br>• Diet resources discussed in this session:<br>o Dietary carbohydrates<br>o Carbohydrate counting<br>o Build your meal – food list<br>o Sample meal plan – 50 g carbs<br>o Drinks, flavourings & extras<br>o Importance of sodium & hydration<br>• Salt pouches provided and participants instructed to measure out and use 1.5 tsp daily to prevent sodium deficiency.<br>• Hardcopy insulin management and diabetes self-monitoring resource pack provided.<br>• Other resources provided/discussed in this session:<br>- Blood glucose monitoring<br>- Ketone self-monitoring<br>- Preparing for your blood test<br>• Participants encouraged to report any adverse events throughout the study.<br>• Participants encouraged to follow-up with their nominated healthcare professional within 7 days to discuss insulin management in more detail.<br>• Review time and dates of all scheduled diet sessions (x6).
**Diabetes education session** (during week 5 or 6)	• Telehealth (Zoom) with study diabetes educator.<br>• Insulin management & diabetes self-monitoring resources reviewed and discussed:<br>- Insulin management considerations

	- Blood glucose monitoring<br>- Self-monitoring ketones<br>- Managing hypoglycaemia<br>- Managing sick days
**Diet session 2** (during week 7)	• Telehealth call (Zoom) with study dietitian.<br>• Review of dietary intake (collect diet history or review Easy Diet Diary tracking) and usual care blood glucose monitoring.<br>• Provide individualised dietary strategies for adhering to the intervention.<br>• Review understanding of information provided in diet session 1.<br>• Diet resources discussed in this session:<br>- Dietary proteins<br>- Dietary fats<br>- Snack ideas<br>• Participants prompted to report any adverse events experienced (self-report) over previous 2 weeks (e.g., hypoglycaemia (<3.5 mmol/L), digestive issues, low energy levels, headaches, etc.)<br>• Participants encouraged to follow-up with their nominated healthcare professional to discuss insulin management.
**Diet session 3** (during week 9)	Telehealth call (Zoom) with study dietitian.<br>• Review of dietary intake (collect diet history or review Easy Diet Diary tracking) and usual care blood glucose monitoring.<br>• Discuss individual preferences, including any challenges or barriers to achieving the recommended carbohydrate target.<br>• **Carb target adjustment (opportunity 1):** If appropriate, negotiate an adapted carbohydrate prescription (i.e., increase or decrease within 25-75 g/day) with the primary goal of achieving pre-specified blood glucose targets (4-6 mmol/L fasting, 4-8 mmol/L 2 hours post meal).<br>• New meal plan provided, if applicable (25 g/day or 60 g/day sample meal plans).<br>• Provide individualised dietary strategies for adhering to the intervention.<br>• Review understanding of information provided in diet session 2.<br>• Participants prompted to report any adverse events experienced (self-report) over previous 2 weeks (e.g., hypoglycaemia (<3.5 mmol/L), digestive issues, low energy levels, headaches, etc.)<br>• Participants encouraged to follow-up with their nominated healthcare professional to discuss insulin management.
**Diet session 4** (during week 11)	Telehealth call (Zoom) with study dietitian.<br>• Review of dietary intake (collect diet history or review Easy Diet Diary tracking) and usual care blood glucose monitoring.<br>• Provide individualised dietary strategies for adhering to the intervention.<br>• Diet resources discussed in this session:<br>- Fast food, takeaway & dining out<br>- Reading food labels<br>- Shopping & food traps<br>- Gut upset on low carb<br>• Data collected on any side effects experienced by participants (self-report) over previous 2 weeks (e.g., hypoglycaemia (<3.5 mmol/L), digestive issues, low energy levels, headaches, etc.).<br>• Participants encouraged to follow-up with their nominated healthcare professional to discuss insulin management.
**Diet session 5**	Telehealth call (Zoom) with study dietitian.

<table>
<tr><td>(during week 13)</td><td>

Review of dietary intake (collect diet history or review Easy Diet Diary tracking) and usual care blood glucose monitoring.
Discuss individual preferences, including any challenges or barriers to achieving the recommended carbohydrate target.
Carb target adjustment (opportunity 2): If appropriate, negotiate an adapted carbohydrate prescription (i.e., increase or decrease within 25-75 g/day) with the primary goal of achieving pre-specified blood glucose targets (4-6 mmol/L fasting, 4-8 mmol/L 2 hours post meal).
New meal plan provided, if applicable (25 g/day or 60 g/day sample meal plans).
Provide individualised dietary strategies for adhering to the intervention.
Review understanding of information provided in diet session 4.
Participants prompted to report any adverse events experienced (self-report) over previous 2 weeks (e.g., severe hypoglycaemia, digestive issues, low energy levels, headaches, etc.)
Participants encouraged to follow-up with their nominated healthcare professional to discuss insulin management.
</td></tr>
<tr><td>Diet session 6
(start of week 16)</td><td>

Review of dietary intake (collect diet history or review Easy Diet Diary tracking) and usual care blood glucose monitoring.
Provide individualised dietary strategies for adhering to the intervention.
Participants prompted to report any adverse events experienced (self-report) over previous 2 weeks (e.g., severe hypoglycaemia, digestive issues, low energy levels, headaches, etc.)
Participants encouraged to follow-up with their nominated healthcare professional to discuss insulin management.
</td></tr>
<tr><td>Clinic visit 5
(start of week 16)
Note – same day as diet session 6</td><td>

Equipment, demonstration and instructions provided for:

CGM/FGM device (sensor(s) and reader) (7-day monitoring)
Insulin logbook (3-day monitoring)
Diet record (Easy Diet Diary mobile app) (3-day monitoring)
Biological samples (Australian Clinical Labs request form provided)

</td></tr>
<tr><td>Clinic visit 6
(end of week 16)</td><td>

Morning of clinic visit 6: Participants attend their nearest Australian Clinical Labs collection centre (fasted) to have biological samples taken. Participants instructed to have a meal and drink fluids after blood draw and before their clinic visit.
Data collected (post-intervention outcomes):

Return/upload CGM/FGM device/data
Return/upload insulin logbook
Return/upload diet record
Anthropometric measurements taken (weight, height, waist circumference and blood pressure)
Questionnaires completed (quality of life, physical activity, diet satisfaction)

End of intervention period – participants thanked for being involved in study and encouraged to follow up with usual healthcare team for all matters.
Provide letter with instructions for follow-up period (Attachment 14)
Provide pre-paid pathology request form (from iMedical) for follow-up HbA1c
Follow-up session scheduled and participants informed they will receive an email with instructions 2 weeks prior.
</td></tr>
<tr><td colspan="2">Follow-up Period</td></tr>
<tr><td>Visit/session</td><td>Outline</td></tr>
</table>

**Email reminder** (start of week 27)	• Participants reminded that they will need to visit an Australian Clinical Labs pathology collection centre ~3 days before their follow-up session.<br>• Brief outline for follow-up call provided.
**Follow-up session** (end of week 28)	Telehealth call (Zoom) with study dietitian.<br>• Data collected (post-control/pre-intervention outcomes):<br>- HbA1c (collected via Australian Clinical Labs and iMedical)<br>- Total daily insulin use (24-hour)<br>- Diet recall (24-hour)<br>- Questionnaires completed (quality of life, diet satisfaction)<br>• End of study – participants thanked for being involved in study and reminded to follow up with usual healthcare team for all matters.

Text message reminders will be sent to participants the day before each visit/session.

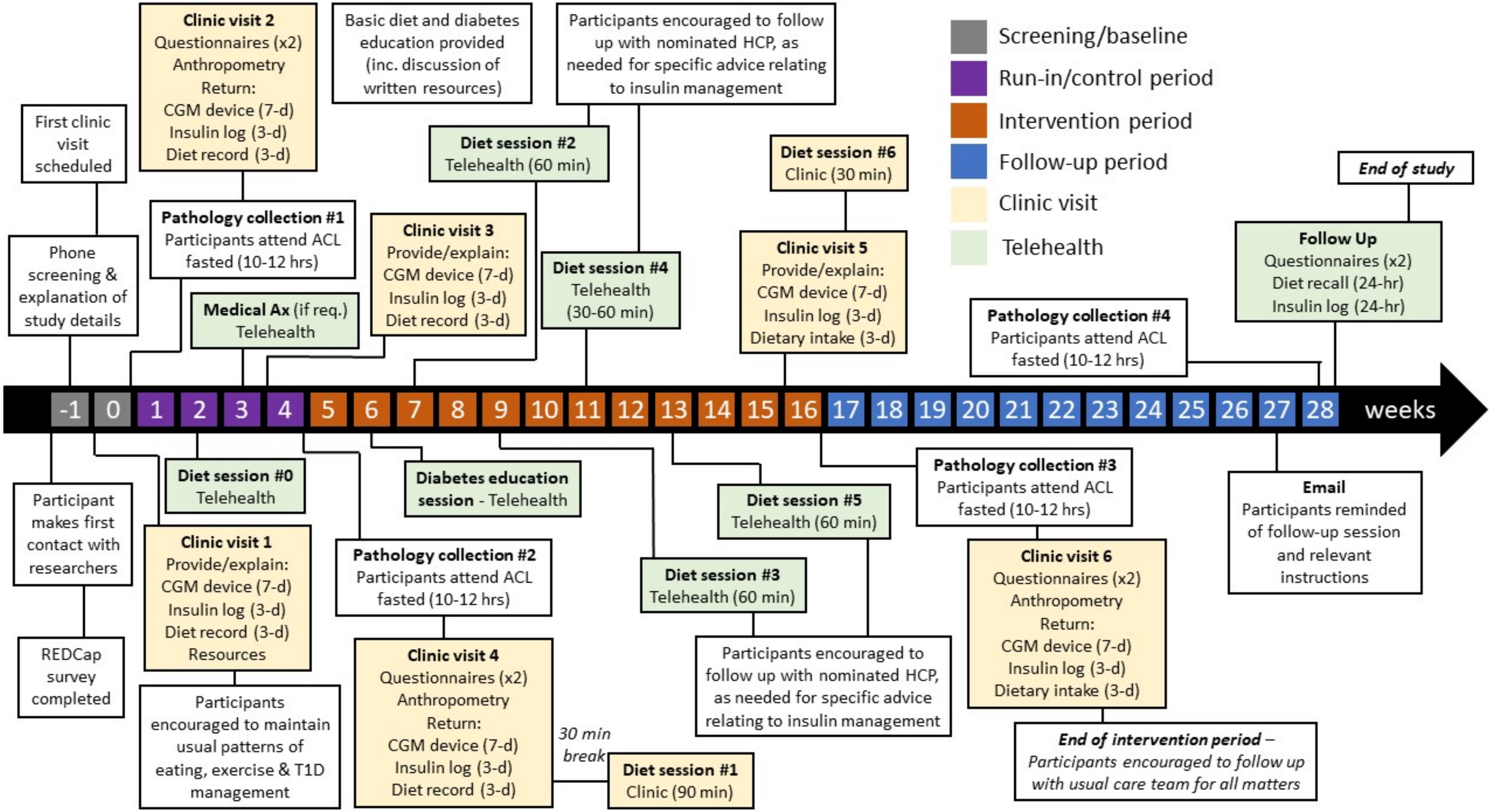

Figure 1. Overview of Contact Schedule for Participants

Abbreviations: CGM, continuous glucose monitor; T1D, type 1 diabetes; HCP, healthcare practitioner; Ax, assessment; req., required; inc., including; d, day; ACL, Australian Clinical Labs Pathology Collection Centre.

Clinic visits may be completed online via Zoom video Conferencing or in person at the Susan Wakil Health Building at the University of Sydney .

Table 2. Study Milestones

Milestone	Description	Time	Date
#1	Study starts & recruitment period begins	0 months	21st June 2021
#2	Recruitment period ends (unless 20 participants recruited prior)	5 months	30th November 2021
#3	The last recruited participant completes intervention period* (if recruited on 30/11/21)	8 months	31st March 2022
#4	The last recruited participant completes follow-up period (final point of data collection)	12 months	30th June 2022
#5	Study ends	12 months	30th June 2022

*based on 18 weeks from completing REDCap survey to completing intervention period.
