# Supplementary material for: Effects of a low-carbohydrate diet in adults with type 1 diabetes management: A single arm non-randomised clinical trial
Source: PLoS One. 2023 Jul 11;18(7):e0288440. doi: 10.1371/journal.pone.0288440 (PMC10335683; doi:10.1371/journal.pone.0288440)
Supplement: S2 Table — Abbreviations: carbs, total dietary carbohydrates; g, grams; tsp, teaspoon; tbs, tablespoon. *Cooked weight; ^natural nut butter (nuts and salt only). Other instructions: If you want to add snacks to this meal plan and your carbohydrate target is 25 g/day, then your snacks should be proteins and/or fats that do not also contain carbs. If you want to increase your portions of proteins and/or fats at meals to reach satiety, you can. (DOCX) [file pone.0288440.s003.docx]

S2 Table. Sample Meal Plan A for Low-Carbohydrate Diet (carbs: 25 g/day)

| **Day** | **Breakfast** | **Lunch** | **Dinner** |
| --- | --- | --- | --- |
| **A** | 2 whole eggs, large; 100 g smoked salmon; ½ avocado (80 g); 1 tbs butter (20 g); 1 small tomato (120 g); black coffee; **carbs: 4 g \| proteins: 39 g \| fats: 46 g.** | 120 g plain yoghurt; 2 tbs almond butter^^^(40g); ½ mandarin (40 g); peppermint tea; **carbs: 13 g \| proteins: 16 g \| fats: 33 g.** | 150 g* beef steak; 50 g cheese, haloumi; 2 tsp olive oil (10 mL); ¼ cucumber (60 g); ½ medium red onion; ½ mandarin (40 g); **carbs: 10 g \| proteins: 53 g \| fats: 33 g.** |
| **B** | 3 whole eggs, large; 50 g cheese, fetta; 8 olives (30 g); ¼ cucumber (60 g); 1 slice watermelon (75 g); green tea; **carbs: 8 g \| proteins: 29 g \| fats: 31 g.** | 100 g* chicken breasts; 50 g cheese, brie; 30 g mixed nuts; 1/3 carrot (40 g); 3 pcs pickles (20 g); **carbs: 10 g \| proteins: 44 g \| fats: 36 g.** | 1 medium lamb chop (150 g*); 1 tbs butter (20 g); ¼ cup green peas (40 g*); 4 baby carrots (65 g*); **carbs: 7 g \| proteins: 43 g \| fats: 47 g.** |
| **C** | 150 g plain yoghurt; 50 g mixed nuts; 2 tsp cream (10 mL); 6 strawberries (70 g); herbal tea; **carbs: 16 g \| proteins: 17 g \| fats: 48 g.** | 100 g* canned tuna; 50 g cream cheese; ½ avocado (80 g); ¼ cucumber (60 g); 3 sheets dried nori (8 g); **carbs: 4 g \| proteins: 34 g \| fats: 31 g.** | 150 g* BBQ chicken; 15 g cheese, parmesan; 15 g pine nuts; 2 tsp olive oil (10 mL); ½ avocado (80 g); 1 cup iceberg lettuce; ¼ capsicum (70 g); **carbs: 4 g \| proteins: 45 g \| fats: 60 g.** |

Abbreviations: carbs, total dietary carbohydrates; g, grams; tsp, teaspoon; tbs, tablespoon.

*Cooked weight; ^^^natural nut butter (nuts and salt only).

Other instructions: If you want to add snacks to this meal plan and your carbohydrate target is 25 g/day, then your snacks should be proteins and/or fats that do not also contain carbs. If you want to increase your portions of proteins and/or fats at meals to reach satiety, you can.
