# Supplementary material for: Effects of a low-carbohydrate diet in adults with type 1 diabetes management: A single arm non-randomised clinical trial
Source: PLoS One. 2023 Jul 11;18(7):e0288440. doi: 10.1371/journal.pone.0288440 (PMC10335683; doi:10.1371/journal.pone.0288440)
Supplement: S3 Table — Abbreviations: carbs, total dietary carbohydrates; g, grams; tsp, teaspoon; tbs, tablespoon. *Cooked weight; ^natural nut butter (nuts and salt only). Other instructions: If you want to add snacks to this meal plan and your carbohydrate target is 50 g/day, then your snacks should be proteins and/or fats that do not also contain carbs. If you want to increase your portions of proteins and/or fats at meals to reach satiety, you can. (DOCX) [file pone.0288440.s004.docx]

S3 Table. Sample Meal Plan B for Low-Carbohydrate Diet (carbs: 50 g/day)

| **Day** | **Breakfast** | **Lunch** | **Dinner** |
| --- | --- | --- | --- |
| **A** | 2 whole eggs, large; 75 g smoked salmon; 1 tbs butter (20 g); 3 asparagus spears; ½ cup mushroom (40 g); 6 cherry tomatoes; small latte (175 mL); **carbs: 11 g \| proteins: 40 g \| fats: 38 g.** | 150 g plain yoghurt; 30 g almonds; 2 tsp cream (10 mL); 10 raspberries (20 g); 15 blueberries (20 g); 20 g 85% dark chocolate; peppermint tea; **carbs: 17 g \| proteins: 17 g \| fats: 43 g.** | 150 g* beef mince; 4 tsp olive oil (20 mL); 40 g tomato paste (2 tbs); ½ cup diced tomato; 1 medium zucchini (zoodles); 8 olives (30 g); 1 tsp Italian herbs; 1 mandarin (75 g); **carbs: 20 g \| proteins: 48 g \| fats: 46 g.** |
| **B** | 150 g plain yoghurt; 30 g mixed nuts; ¼ cup dried coconut; 4 tsp rolled oats (7 g); 6 strawberries (70 g); herbal tea; **carbs: 18 g \| proteins: 15 g \| fats: 49 g.** | 2 whole eggs, large; 80 g cheese, haloumi; 2 tsp olive oil (10 mL); ½ avocado (80 g); ½ cup mushrooms (40 g); 1 tomato (150 g); small latte (175 mL); **carbs: 15 g \| proteins: 38 g \| fats: 47 g.** | 120 g* chicken thighs; 30 g cheese, parmesan; 1 tbs butter (20 g); ½ cup broccoli (75 g*); 120 g* pumpkin; 3 beetroot slices (60 g); **carbs: 15 g \| proteins: 46 g \| fats: 36 g.** |
| **C** | Smoothie [120 g plain yoghurt; 2 tbs peanut butter^^^(40 g); 2 tsp cream (10 mL); ½ cup berries (80 g); 75 mL cow’s milk; water + ice (as required)]; **carbs: 19 g \| proteins: 19 g \| fats: 40 g.** | 100 g* canned tuna; 50 g cheese, fetta; 4 tsp olive oil (20 mL); 1 cup leafy greens; 3 beetroot slices (60 g); 6 cherry tomatoes (50 g); 40 g sauerkraut; 20 g 85% dark chocolate; **carbs: 12 g \| proteins: 38 g \| fats: 42 g.** | 150 g* salmon fillet; 1 tbs butter (20 g); ½ cup broccoli (75 g*); 120 g* pumpkin; ¼ cucumber (60 g); 1 slice watermelon (75 g); fresh mint leaves (10 g); **carbs: 16 g \| proteins: 49 g \| fats: 52 g.** |

Abbreviations: carbs, total dietary carbohydrates; g, grams; tsp, teaspoon; tbs, tablespoon.

*Cooked weight; ^^^natural nut butter (nuts and salt only).

Other instructions: If you want to add snacks to this meal plan and your carbohydrate target is 50 g/day, then your snacks should be proteins and/or fats that do not also contain carbs. If you want to increase your portions of proteins and/or fats at meals to reach satiety, you can.
