# Supplementary material for: Effects of a low-carbohydrate diet in adults with type 1 diabetes management: A single arm non-randomised clinical trial
Source: PLoS One. 2023 Jul 11;18(7):e0288440. doi: 10.1371/journal.pone.0288440 (PMC10335683; doi:10.1371/journal.pone.0288440)
Supplement: S5 Table — Data presented for n = 15 with exclusions applied to the raw data (days that had <80% complete values were excluded) and participants with <3 days of complete data were excluded (n = 1). Data presented as means and standard deviations or medians and interquartile ranges (indicated by ^). *P<0.025, †P<0.01, and ‡P<0.001; indicates significantly different from post-control. (DOCX) [file pone.0288440.s006.docx]

S5 Table. Glycaemic Variability Outcomes with Exclusions (n=15)

|  | **Pre-control** | **Post-control** | **Post-intervention** |
| --- | --- | --- | --- |
| **Time in range** (%) | 53.7 (15.9) | 58.8 (18.5) | 73.9 (18.6)^‡^ |
| **Mean glucose** (mmol/L) | 9.7 (1.6) | 9.3 (1.6) | 8.2 (1.7)^‡^ |
| **MAGE** | 8.0 (1.8) | 7.0 (1.0) | 5.7 (1.7)^†^ |
| **Standard deviation** | 3.3 (0.6)^*^ | 2.8 (0.6) | 2.2 (0.6)^‡^ |

Data presented for n=15 with exclusions applied to the raw data (days that had <80% complete values were excluded) and participants with <3 days of complete data were excluded (n=1).

Data presented as means and standard deviations or medians and interquartile ranges (indicated by ^).

^*^P<0.025, ^†^P<0.01, and ^‡^P<0.001; indicates significantly different from post-control.
