# Supplementary material for: Effects of a low-carbohydrate diet in adults with type 1 diabetes management: A single arm non-randomised clinical trial
Source: PLoS One. 2023 Jul 11;18(7):e0288440. doi: 10.1371/journal.pone.0288440 (PMC10335683; doi:10.1371/journal.pone.0288440)
Supplement: S6 Table — Data presented for n = 20 (intention to treat). Data presented as means and standard deviations or medians and interquartile ranges (indicated by ^). * = P<0.025 between timepoints (post-control and pre-control or post-intervention and post-control). Abbreviations–int, intervention; kJ, kilojoules, Cal, Calories; g, grams; TEI, total energy intake. (DOCX) [file pone.0288440.s007.docx]

S6 Table. Dietary intake for participants with type 1 diabetes during control and intervention periods (intention-to-treat).

|  | **Pre-control** | **Post-control** | **Post-int.** |
| --- | --- | --- | --- |
| **Total energy** (kJ/day) | 10018.4 (2588.9) | 9687.6 (2291.4) | 8574.3 (2625.2)* |
| **Total energy** (Cal/day) | 2393.4 (618.5) | 2314.3 (547.3) | 2048.4 (627.2)* |
| **Carbohydrates** (g/day) | 224.3 (74.8) | 215.0 (71.9) | 94.6 (88.5)* |
| **Carbohydrates** (%TEI) | 35.0 (6.5) | 35.5 (8.3) | 16.8 (12.0)* |
| **Proteins** (g/day) | 105.3 (26.3) | 109.5 (33.5) | 121.2 (27.9) |
| **Proteins** (%TEI) | 18.3 (3.9) | 19.4 (4.7) | 24.9 (4.7)* |
| **Fats** (g/day) | 98.5 (26.0) | 100.0 (31.3) | 127.5 (50.7)* |
| **Fats** (%TEI) | 36.6 (5.2) | 38.0 (6.2) | 54.9 (10.8)* |
| **Saturated fats** (g/day) | 37.8 (14.1) | 36.5 (12.9) | 51.0 (30.7)* |
| **Saturated fats** (%TEI) | 14.2 (4.2) | 13.8 (3.2) | 22.6 (7.3)* |
| **Fibres** (g/day) | 24.1 (7.0) | 23.1 (7.3) | 20.1 (8.0) |
| **Alcohol** (g/day)^ | 15.0 (31.0) | 2.5 (22.0) | 0.0 (4.0)* |

Data presented for n=20 (intention to treat).

Data presented as means and standard deviations or medians and interquartile ranges (indicated by ^).

*=P<0.025 between timepoints (post-control and pre-control or post-intervention and post-control).

Abbreviations – int, intervention; kJ, kilojoules, Cal, Calories; g, grams; TEI, total energy intake.
