# Supplementary material for: Effects of a low-carbohydrate diet in adults with type 1 diabetes management: A single arm non-randomised clinical trial
Source: PLoS One. 2023 Jul 11;18(7):e0288440. doi: 10.1371/journal.pone.0288440 (PMC10335683; doi:10.1371/journal.pone.0288440)
Supplement: S7 Table — Data presented for n = 20 (intention to treat), except systolic BP and diastolic BP (n = 19) due to missing data at >1 timepoints. Data presented as means and standard deviations or medians and interquartile ranges (indicated by ^). * = P<0.025 between timepoints (post-control and pre-control or post-int and post-control). #A lower score implies a more satisfactory quality of life. Abbreviations–HbA1c, glycated haemoglobin; MAGE, mean amplitude of glycaemic excursions; hypo, hypoglycaemia; BP, blood pressure; HDL, high density lipoprotein; LDL, low density lipoprotein. (DOCX) [file pone.0288440.s008.docx]

S7 Table. Main clinical outcomes for participants with type 1 diabetes during control and intervention periods (intention-to-treat).

|  | **Pre-control** | **Post-control** | **Post-intervention** |
| --- | --- | --- | --- |
| ***Glycaemic Control*** |  |  |  |
| **HbA1c** (%)^ | 7.8 (0.6) | 7.9 (0.8) | 7.5 (1.1)* |
| **Fasting blood glucose** (mmol/L) | 9.0 (3.5) | 8.9 (3.5) | 6.8 (3.3)* |
| **Time in range** (%) | 54.8 (16.3) | 56.2 (19.1) | 68.7 (23.2)* |
| **Mean glucose** (mmol/L) | 9.8 (1.8) | 9.7 (2.0) | 8.6 (2.3)* |
| **MAGE** (mmol/L) | 8.0 (2.0) | 7.3 (1.1) | 5.7 (1.7)* |
| **Standard deviation of blood glucose** | 3.1 (0.6) | 2.8 (0.5) | 2.2 (0.6)* |
| **Hypo frequency** (episodes/day)^ | 0.3 (0.3) | 0.3 (0.6) | 0.3 (0.7) |
| **Total daily insulin** (units/day) | 63.0 (21.2) | 62.2 (22.1) | 49.3 (19.2)* |
| ***Anthropometry*** |  |  |  |
| **Body mass index** (kg/m^2^) | 29.8 (5.9) | 29.9 (6.0) | 29.3 (5.6)* |
| **Body weight** (kg) | 87.7 (18.2) | 87.7 (18.4) | 86.0 (17.2)* |
| **Waist circumference** (cm) | 99.4 (16.0) | 99.4 (15.2) | 97.3 (14.5) |
| **Systolic BP** (mmHg) | 125.5 (12.3) | 130.2 (11.8) | 130.9 (15.8) |
| **Diastolic BP** (mmHg) | 78.6 (6.9) | 76.9 (9.8) | 77.8 (8.8) |
| ***Lipids*** |  |  |  |
| **Total cholesterol** (mmol/L) | 4.6 (0.8) | 4.5 (0.7) | 4.8 (1.1) |
| **HDL cholesterol** (mmol/L) | 1.5 (0.5) | 1.5 (0.5) | 1.6 (0.4) |
| **LDL cholesterol** (mmol/L)^ | 2.5 (0.9) | 2.4 (0.7) | 2.5 (1.5) |
| **Triglycerides** (mmol/L)^ | 0.9 (0.4) | 0.9 (0.5) | 0.9 (0.3) |
| **Diet satisfaction** (% satisfied)^ | 85.7 (39.3) | 71.4 (50.0) | 100.0 (50.0) |
| **Diabetes quality of life^#^** | 35.2 (7.4) | 34.1 (6.9) | 31.3 (8.3)* |

Data presented for n=20 (intention to treat), except SBP and DBP (n=19) due to missing data at >1 timepoints.

Data presented as means and standard deviations or medians and interquartile ranges (indicated by ^).

*=P<0.025 between timepoints (post-control and pre-control or post-int and post-control).

^#^A lower score implies a more satisfactory quality of life.

Abbreviations – HbA1c, glycated haemoglobin; MAGE, mean amplitude of glycaemic excursions; hypo, hypoglycaemia; BP, blood pressure; HDL, high density lipoprotein; LDL, low density lipoprotein.
