# Supplementary material for: Effects of a low-carbohydrate diet in adults with type 1 diabetes management: A single arm non-randomised clinical trial
Source: PLoS One. 2023 Jul 11;18(7):e0288440. doi: 10.1371/journal.pone.0288440 (PMC10335683; doi:10.1371/journal.pone.0288440)
Supplement: S8 Table — Data presented for n = 20 (intention to treat), except uric acid (n = 19) due to missing data at >1 timepoints. Data presented as means and standard deviations or medians and interquartile ranges (indicated by ^). * = P<0.025 between timepoints (post-control and pre-control or post-intervention and post-control). (DOCX) [file pone.0288440.s009.docx]

S8 Table. Additional outcomes for participants with type 1 diabetes during control and intervention periods (intention-to-treat).

|  | **Pre-control** | **Post-control** | **Post-intervention** |
| --- | --- | --- | --- |
| **Sodium** (mmol/L) | 139.1 (2.4) | 139.1 (2.2) | 138.9 (1.9) |
| **Potassium** (mmol/L) | 4.4 (0.3) | 4.4 (0.2) | 4.4 (0.2) |
| **Chloride** (mmol/L) | 104.4 (3.4) | 104.6 (2.7) | 105.2 (3.3) |
| **Bicarbonate** (mmol/L) | 27.5 (2.8) | 26.8 (2.8) | 26.9 (2.4) |
| **Urea** (mmol/L) | 5.6 (1.5) | 5.6 (1.5) | 5.7 (1.4) |
| **Creatinine** (umol/L) | 83.2 (11.8) | 80.7 (13.0) | 78.8 (10.3) |
| **eGFR** (mL/min/1.73m^2^)^ | 88.0 (16.0) | 89.5 (12.0) | 89.5 (8.0) |
| **Calcium** (mmol/L) | 2.4 (0.1) | 2.4 (0.1) | 2.4 (0.1) |
| **Corrected calcium** (mmol/L) | 2.4 (0.1) | 2.4 (0.1) | 2.4 (0.1) |
| **Phosphate** (mmol/L) | 1.1 (0.2) | 1.1 (0.3) | 1.1 (0.2) |
| **Uric acid** (mmol/L)^ | 0.3 (0.1) | 0.3 (0.1) | 0.3 (0.1) |
| **Total protein** (g/L) | 69.1 (3.5) | 68.7 (3.6) | 69.1 (3.5) |
| **Albumin** (g/L) | 39.5 (2.5) | 39.1 (3.3) | 39.4 (3.2) |
| **Alkaline phosphatase** (U/L) | 74.3 (24.5) | 74.7 (24.2) | 71.4 (20.5) |
| **Bilirubin** (umol/L)^ | 11.0 (3.0) | 8.5 (5.0) | 8.5 (6.0) |
| **Gamma GT** (U/L)^ | 18.0 (11.0) | 17.0 (14.0) | 15.5 (12.0) |
| **AST** (U/L) | 22.4 (6.4) | 21.2 (4.6) | 22.3 (6.1) |
| **ALT** (U/L)^ | 21.5 (12.0) | 21.5 (16.0) | 22.5 (15.0) |
| **Globulin** (g/L) | 30.0 (3.9) | 29.7 (3.9) | 29.8 (3.9) |
| **Magnesium** (mmol/L) | 0.8 (0.1) | 0.8 (0.1) | 0.8 (0.1) |
| **Creatine kinase** (U/L)^ | 104.0 (124.0) | 116.5 (75.0) | 173.0 (163.0) |

Data presented for n=20 (intention to treat), except uric acid (n=19) due to missing data at >1 timepoints.

Data presented as means and standard deviations or medians and interquartile ranges (indicated by ^).

*=P<0.025 between timepoints (post-control and pre-control or post-intervention and post-control).
