# Supplementary material for: Effects of a low-carbohydrate diet in adults with type 1 diabetes management: A single arm non-randomised clinical trial
Source: PLoS One. 2023 Jul 11;18(7):e0288440. doi: 10.1371/journal.pone.0288440 (PMC10335683; doi:10.1371/journal.pone.0288440)
Supplement: S9 Table — Data presented as means and standard deviations or medians and interquartile ranges (indicated by ^). * = P<0.025 between timepoints (post-control and pre-control or post-intervention and post-control). Abbreviations–PAL, physical activity level; MET, metabolic equivalent of task; min, minutes, ITT, intention to treat. (DOCX) [file pone.0288440.s010.docx]

S9 Table. Total physical activity and time spent sitting for participants with type 1 diabetes during control and intervention periods.

|  | **Pre-control** | **Post-control** | **Post-int.** |
| --- | --- | --- | --- |
| **PAL (MET min/day)** |  |  |  |
| Completers (n=16)^ | 2824.5 (3878.3) | 2407.5 (3581.5) | 3560.5 (6117.0) |
| ITT (n=20)^ | 2973.5 (3878.3) | 2407.5 (3442.0) | 3335.5 (4960.3) |
| **Time sitting (min/day)** |  |  |  |
| Completers (n=16) | 396.4 (192.9) | 405.0 (226.0) | 379.3 (204.3) |
| ITT (n=20) | 389.5 (193.9) | 395.6 (218.5) | 375.0 (200.1) |

Data presented as means and standard deviations or medians and interquartile ranges (indicated by ^).

*=P<0.025 between timepoints (post-control and pre-control or post-intervention and post-control).

Abbreviations – PAL, physical activity level; MET, metabolic equivalent of task; min, minutes, ITT, intention to treat.
