# Supplementary material for: Effects of a low-carbohydrate diet in adults with type 1 diabetes management: A single arm non-randomised clinical trial
Source: PLoS One. 2023 Jul 11;18(7):e0288440. doi: 10.1371/journal.pone.0288440 (PMC10335683; doi:10.1371/journal.pone.0288440)
Supplement: S10 Table — *data taken from screening survey. (DOCX) [file pone.0288440.s011.docx]

S10 Table. Use of medications other than insulin by participants with type 1 diabetes at baseline.*

| **Type/Name of Medication** | **Completers (n=16)** | **Dropouts (n=14)** |
| --- | --- | --- |
| Cholesterol-lowering | 8 |  |
| Oral anti-glycaemic | 1 |  |
| Anti-hypertensive | 4 | 1 |
| Anti-convulsant (Primidone) | 1 |  |
| Anti-rheumatic (Plaquenil) | 1 |  |
| Immune system suppressant (Methotrexate) | 1 |  |
| Anti-depressant (Sertraline, Zoloft) | 1 | 1 |
| Thyroxine | 1 |  |
| Salazopryrin |  | 1 |
| Oral contraceptive | 2 |  |
| Menopausal treatment (Livial, Prometrium, Estrogel) | 2 |  |

*data taken from screening survey
