# Supplementary material for: Effects of a low-carbohydrate diet in adults with type 1 diabetes management: A single arm non-randomised clinical trial
Source: PLoS One. 2023 Jul 11;18(7):e0288440. doi: 10.1371/journal.pone.0288440 (PMC10335683; doi:10.1371/journal.pone.0288440)
Supplement: S11 Table — Data presented for n = 16 (completers), except calcium (n = 15), corrected calcium (n = 15), phosphate (n = 15), uric acid (n = 15), magnesium (n = 15), creatine kinase (n = 15), and body weight (n = 13) due to missing data at ≥1 timepoint. Data for pre-control, post-control, and post-intervention timepoints presented as means and standard deviations or medians and interquartile ranges (indicated by ^). * = P<0.025. #A lower score implies a more satisfactory quality of life. (DOCX) [file pone.0288440.s012.docx]

S11 Table. Additional outcomes for participants with type 1 diabetes during control and intervention periods (n=16).

|  | **Pre-control** | **Post-control** | **Post-intervention** |
| --- | --- | --- | --- |
| **Sodium** (mmol/L) | 139.8 (1.9) | 139.7 (1.8) | 139.4 (1.4) |
| **Potassium** (mmol/L) | 4.4 (0.2) | 4.3 (0.2) | 4.4 (0.2) |
| **Chloride** (mmol/L)^ | 106.0 (3.0) | 105.5 (4.0) | 106.0 (5.0) |
| **Bicarbonate** (mmol/L) | 27.3 (2.8) | 26.9 (3.0) | 27.0 (2.7) |
| **Urea** (mmol/L) | 5.9 (1.5) | 5.7 (1.4) | 5.8 (1.3) |
| **Creatinine** (umol/L) | 85.8 (10.4) | 82.9 (11.8) | 80.5 (8.3) |
| **eGFR** (mL/min/1.73m^2^)^ | 84.5 (17.0) | 89.0 (15.0) | 88.5 (8.0) |
| **Calcium** (mmol/L) | 2.4 (0.1) | 2.4 (0.1) | 2.4 (0.1) |
| **Corrected calcium** (mmol/L) | 2.4 (0.1) | 2.4 (0.1) | 2.4 (0.1) |
| **Phosphate** (mmol/L) | 1.1 (0.2) | 1.1 (0.3) | 1.1 (0.2) |
| **Uric acid** (mmol/L)^ | 0.3 (0.1) | 0.3 (0.1) | 0.3 (0.1) |
| **Total protein** (g/L) | 69.1 (3.6) | 68.6 (3.7) | 69.1 (3.6) |
| **Albumin** (g/L) | 38.9 (2.4) | 38.6 (3.2) | 38.9 (3.2) |
| **Alkaline phosphatase** (U/L) | 73.3 (22.0) | 75.3 (24.0) | 71.2 (19.0) |
| **Bilirubin** (umol/L) | 11.4 (5.9) | 11.2 (5.1) | 10.8 (5.8) |
| **Gamma GT** (U/L)^ | 19.5 (13.0) | 17.5 (16.0) | 16.0 (12.0) |
| **AST** (U/L) | 22.0 (7.1) | 21.1 (5.0) | 22.5 (6.7) |
| **ALT** (U/L) | 23.6 (14.9) | 22.2 (8.9) | 22.8 (8.7) |
| **Globulin** (g/L) | 30.2 (4.2) | 30.1 (4.0) | 30.2 (4.1) |
| **Magnesium** (mmol/L) | 0.8 (0.1) | 0.8 (0.1) | 0.8 (0.1) |
| **Creatine kinase** (U/L)^ | 101.0 (156.0) | 119.0 (69.0) | 208.0 (176.0)* |

Data presented for n=16 (completers), except calcium (n=15), corrected calcium (n=15), phosphate (n=15), uric acid (n=15), magnesium (n=15), creatine kinase (n=15), and body weight (n=13) due to missing data at ≥1 timepoint.

Data for pre-control, post-control, and post-intervention timepoints presented as means and standard deviations or medians and interquartile ranges (indicated by ^).

*=P<0.025 between timepoints (post-control and pre-control or post-control and post-intervention).

^#^A lower score implies a more satisfactory quality of life.
