# Supplementary material for: Effects of a low-carbohydrate diet in adults with type 1 diabetes management: A single arm non-randomised clinical trial
Source: PLoS One. 2023 Jul 11;18(7):e0288440. doi: 10.1371/journal.pone.0288440 (PMC10335683; doi:10.1371/journal.pone.0288440)
Supplement: Attachment — Submitted filename: Response to Reviewer Comments_Turton et al R2 MASTER.pdf [file pone.0288440.s014.pdf]

THE UNIVERSITY OF SYDNEY

Date: 10th May 2023
Jessica.turton@sydney.edu.au
Faculty of Medicine & Health, Susan Wakil Health Building
University of Sydney

Response to Reviewer Comments

Reviewer 1

Comment 1: *Overall the authors have made good progress with amending the paper, though I thought that the responses to the extensive reviewer comments could have been more elaborate and substantive. I think it is wrong to conclude, as the authors have done, that "This study demonstrated that a professionally supported LC diet improved markers of blood glucose control and quality of life with reduced exogenous insulin requirements and no evidence of increased risk of hypoglycaemia or ketoacidosis" - If it helps the authors, I would suggest re-writing this to state that "These preliminary findings suggest that a professionally supported LC diet may lead to improvements in markers of blood glucose control and quality of life with reduced exogenous insulin requirements and no evidence of increased risk of hypoglycaemia or ketoacidosis. However a randomised controlled trial would need to be undertaken to demonstrate the efficacy of this intervention to influence these clinical outcomes. Given the potential benefits of this intervention, such a trial is warranted." For me, amending the level of inference around clinical practice that the authors have made in the conclusions would be essential before this paper would be suitable for publication.*

Response: The authors thank this reviewer for their thorough review and constructive comments. It is confirmed that the conclusion has now been updated in the Abstract and the main text Conclusions.

The respective section of the Abstract (page 2, lines 34-39) now reads as:

"These preliminary findings suggest that a professionally supported LC diet may lead to improvements in markers of blood glucose control and quality of life with reduced exogenous insulin requirements and no evidence of increased hypoglycaemia or ketoacidosis risk in adults with T1D. Given the potential benefits of this intervention, larger, longer-term randomised controlled trials are warranted to confirm these findings."

The respective section of the main text Conclusions (page 28, lines 556-561) now reads as:

"These preliminary findings suggest that a professionally supported LC diet may improve markers of blood glucose control and quality of life with reduced exogenous insulin requirements and no evidence of increased risk of hypoglycaemia or

ketoacidosis in adults with T1D. Given the potential benefits of this intervention, larger, longer-term randomised controlled trials are needed to confirm these findings and examine clinical endpoints to better demonstrate the efficacy of LC diets in T1D management."

Reviewer 2

(No comments to address)

Reviewer 3

Comment 1: *This is a well-designed study and well written manuscript on the effects of a LC on glycemic control and other health outcomes in type 1 diabetes. The findings of the study are in line with previous observational studies and interventions that showed improvements in glycemic and other health outcomes following a LC diet in type 1 diabetes in adults. Nevertheless, the intervention is innovative given that it was administered remotely by professionals, which also makes it practical and relevant in the current times with the widespread use of telehealth. The authors have adequately addressed the comments made by the reviewers in the first round of review, especially pertaining to the statistical analyses and acknowledging the study's limitations regarding the small sample size, the short-term testing of the intervention and outcomes and the quasi-experimental design.*

Response: The authors thank this reviewer for their thoughtful and positive review of our manuscript.

Comment 2: *Authors need to add to the discussion that longer term studies are needed, not only to test the long-term effects of LC in type 2 diabetes [correction: type 1 diabetes], but also to test the LC diet sustainability and its subsequent effects on improved caloric intake and weight loss, which could be mediating factors for improved health outcomes. This is especially important, given that LC diets are known to induce weight loss in the short term only, and that their effects on weight loses its significance in the longer term, compared to other diets.*

Response: The authors agree that additional clinical trials are needed to better understand the long-term effects of LC diets on weight management for T1D, as well as to understand the effect(s) of LC diets on T1D management outcomes during energy balance, given that weight loss has been associated with improved health outcomes, including glycaemic control.

The relevant paragraph of the discussion (page 24, lines 458-462) has been updated to include the following:

“Future studies should examine the long-term effects (>12 weeks) of LC diets on weight management and diet sustainability in T1D. Further, randomised controlled trials which aim to control for the potential effect(s) of weight loss on glycaemic control outcomes under energy balance conditions are also worthy of consideration.”

Comment 3: *Additionally, authors need to stress in the discussion on the importance of diet quality when recommending a LC diet for type 1 diabetes management, like emphasizing whole foods and healthy protein and fat sources rather than processed sources, as described in the study protocol (Ref 30: Turton JL, Brinkworth GD, Parker HM, Lee K, Lim D, Rush A, et al. Effects of a low-carbohydrate diet in adults with type 1 diabetes: an interventional study protocol. 2021.8(3):11. Epub 2021-07-22. doi: 10.18203/2349-3259.ijct20212846.), which might also be another contributing factor for the favorable health outcomes.*

Response: The authors agree with the need to acknowledge the potential effect(s) of improving diet quality on T1D management outcomes in this study. As such, a paragraph discussing this has now been added to the discussion.

The relevant paragraph of the discussion (page 27, lines 541-552) reads as:

“In the present study, the LC diet intervention prescribed the consumption of minimally-processed whole foods and to minimise the intake of ultra-processed foods. However, due to the preliminary nature of this single arm study that did not assess diet quality, it is difficult to determine the specific effect(s) of changes in diet quality on the favourable metabolic changes observed. Prioritisation of minimally-processed foods has been identified as a core feature of safe and effective LC diet interventions used in T2D management.(32) In addition, common health-promoting dietary patterns, such as the Mediterranean diet, also prioritise consumption of minimally-processed foods such as dairy, nuts, seeds, legumes, meat, fish, and eggs, while limiting ultra-processed foods.(92,93) Future randomised controlled studies should aim to control for the potential impact(s) of diet quality on T1D management outcomes when comparing LC diets with diets higher in carbohydrates. Nevertheless, it would be prudent to consider diet quality in the design and delivery of LC diets in clinical practice.”

[END OF REPORT]
